# Targeting gut microbiota and immune crosstalk: potential mechanisms of natural products in the treatment of atherosclerosis

**DOI:** 10.3389/fphar.2023.1252907

**Published:** 2023-09-01

**Authors:** Jinpeng Jing, Jing Guo, Rui Dai, Chaojun Zhu, Zhaohui Zhang

**Affiliations:** ^1^ Graduate School, Tianjin University of Traditional Chinese Medicine, Tianjin, China; ^2^ Institute of TCM Ulcers, Tianjin University of Traditional Chinese Medicine, Tianjin, China; ^3^ Surgical Department of Traditional Chinese Medicine, Second Affiliated Hospital of Tianjin University of Traditional Chinese Medicine, Tianjin, China

**Keywords:** gut microbiota, immune cells, natural products, atherosclerosis, probiotics

## Abstract

Atherosclerosis (AS) is a chronic inflammatory reaction that primarily affects large and medium-sized arteries. It is a major cause of cardiovascular disease and peripheral arterial occlusive disease. The pathogenesis of AS involves specific structural and functional alterations in various populations of vascular cells at different stages of the disease. The immune response is involved throughout the entire developmental stage of AS, and targeting immune cells presents a promising avenue for its treatment. Over the past 2 decades, studies have shown that gut microbiota (GM) and its metabolites, such as trimethylamine-N-oxide, have a significant impact on the progression of AS. Interestingly, it has also been reported that there are complex mechanisms of action between GM and their metabolites, immune responses, and natural products that can have an impact on AS. GM and its metabolites regulate the functional expression of immune cells and have potential impacts on AS. Natural products have a wide range of health properties, and researchers are increasingly focusing on their role in AS. Now, there is compelling evidence that natural products provide an alternative approach to improving immune function in the AS microenvironment by modulating the GM. Natural product metabolites such as resveratrol, berberine, curcumin, and quercetin may improve the intestinal microenvironment by modulating the relative abundance of GM, which in turn influences the accumulation of GM metabolites. Natural products can delay the progression of AS by regulating the metabolism of GM, inhibiting the migration of monocytes and macrophages, promoting the polarization of the M2 phenotype of macrophages, down-regulating the level of inflammatory factors, regulating the balance of Treg/Th17, and inhibiting the formation of foam cells. Based on the above, we describe recent advances in the use of natural products that target GM and immune cells crosstalk to treat AS, which may bring some insights to guide the treatment of AS.

## 1 Introduction

Atherosclerosis (AS) is a chronic inflammatory disease of arterial wall thickening or functional degeneration associated with disorders of lipid metabolism and immune inflammation and is the major pathological basis of cardiovascular disease, stroke, and peripheral arterial occlusive disease (Libby, 2021). Currently, cardiovascular disease is the leading cause of death and disability for patients worldwide (Roth et al., 2020) and is a worldwide problem shared by modern society (Libby et al., 2019). According to statistics, the number of people with cardiovascular disease worldwide alone has almost doubled from 1990 to 2019, from 271 million to 523 million cases (Roth et al., 2020). Age is the largest risk factor for AS-related diseases, for example, the incidence of peripheral arterial occlusive disease is only 1% in people aged 40–49 years, whereas it is AS high as 15% in people aged 70 years and older (Aday and Matsushita, 2021). Gender is also an influencing factor in AS-related diseases. The incidence of coronary artery disease in premenopausal women is only 10%–30% of that in men, and the risk in postmenopausal women is comparable to that in men (Huang et al., 2020). By age 70, the incidence of myocardial infarction in women catches up with that in men (Man et al., 2020). With the development of the social economy and the change in dietary structure, the incidence of AS continues to rise, which further raises people’s concerns about AS. The first-line drugs for the treatment of AS, such as statins and antiplatelet drugs, have shown good efficacy, but there are some side effects (Satny et al., 2021). In addition, many patients suffer from recurrent clinical events despite achieving target therapeutic levels of low-density lipoprotein (LDL) (Doran, 2022). Therefore, the active search for new ways to treat AS is a race faced by all clinicians.

Gut microbiota (GM), known as the “second genome” of the human body, has evolved with humans for millions of years and plays an important role in regulating the development of human diseases (Chen et al., 2021). GM is interdependent and inter-constrained with the environment and host, participating in various physiological processes such as absorption, digestion, metabolism, and immunity to dynamically maintain the micro-ecological balance of the body (Adak and Khan, 2019). Everyone has a unique GM. The composition and proportion of GM are closely related to the occurrence of AS (Liu et al., 2019). Meanwhile, intermediates of GM metabolism, such as trimethylamine oxides (TMAO) and short-chain fatty acids (SCFAs), can act on many cells and molecules in the development of AS and play a disease-modifying role (Cao et al., 2023). When the quantity or quality of GM changes, the microecological balance of the body is disrupted, and the GM is disordered or dysregulated, which can lead to the occurrence and development of a variety of diseases. There is growing evidence for potential mechanisms of GM and immune response in AS-related diseases, which may provide new targets for the treatment of AS. In pathological states, GM (especially conditionally pathogenic bacteria) and their metabolites stimulate mucosal immune cell activation, hyperactivate the mucosal immune response, and mediate the production of multiple inflammation-associated cytokines (Neurath, 2019; Graham and Xavier, 2020). This link between the GM and the immune system will help to better understand the pathogenesis of AS and facilitate the development of novel therapeutic agents.

Due to their wide range of targets and low toxic effects, natural products are currently an important route for new drug discovery. Accumulating research evidence suggests that natural products can inhibit the progression of AS (Duan et al., 2021; Zhang et al., 2021). In recent years, there have been many studies on the role of GM and immune crosstalk in AS. So, could the GM play a role as a “bridge” between natural products and the immune system to inhibit the development of AS? The answer is yes. At present, some studies have provided support. Therefore, natural products with anti-AS properties may improve AS by modulating GM and immune system crosstalk, which opens up new avenues for the research and treatment of AS. We searched PubMed and Google Scholar using the keywords such as “gut microbiota”, “natural products”, “herbal medicine”, “monocytes”, “macrophages”, “dendritic cells”, “T cells”, “B cells”, and “mast cells”, and the search time was set to nearly 10 years. This article reviews the influence and mechanisms of GM and immunity in AS and summarizes the natural products that have been used as targets for treatment, to provide new insights into the prevention and treatment of AS.

## 2 Role of immune cells in AS

The innate immune response mainly plays a role in removing dead cells, presenting antigens, and protecting the host from external microorganisms (Zarif et al., 2016). Monocytes and macrophages are key effectors and regulators in the innate immune as well as inflammatory response (Moriya, 2019). As a bridge between innate and adaptive immunity, dendritic cells (DCs) initiates and regulate highly pathogen-specific adaptive immune responses and are central to the development of immune memory and tolerance (Liu et al., 2021). T and B lymphocytes recognize the presented antigens and dominate the development of adaptive immune responses.

**FIGURE 1:**
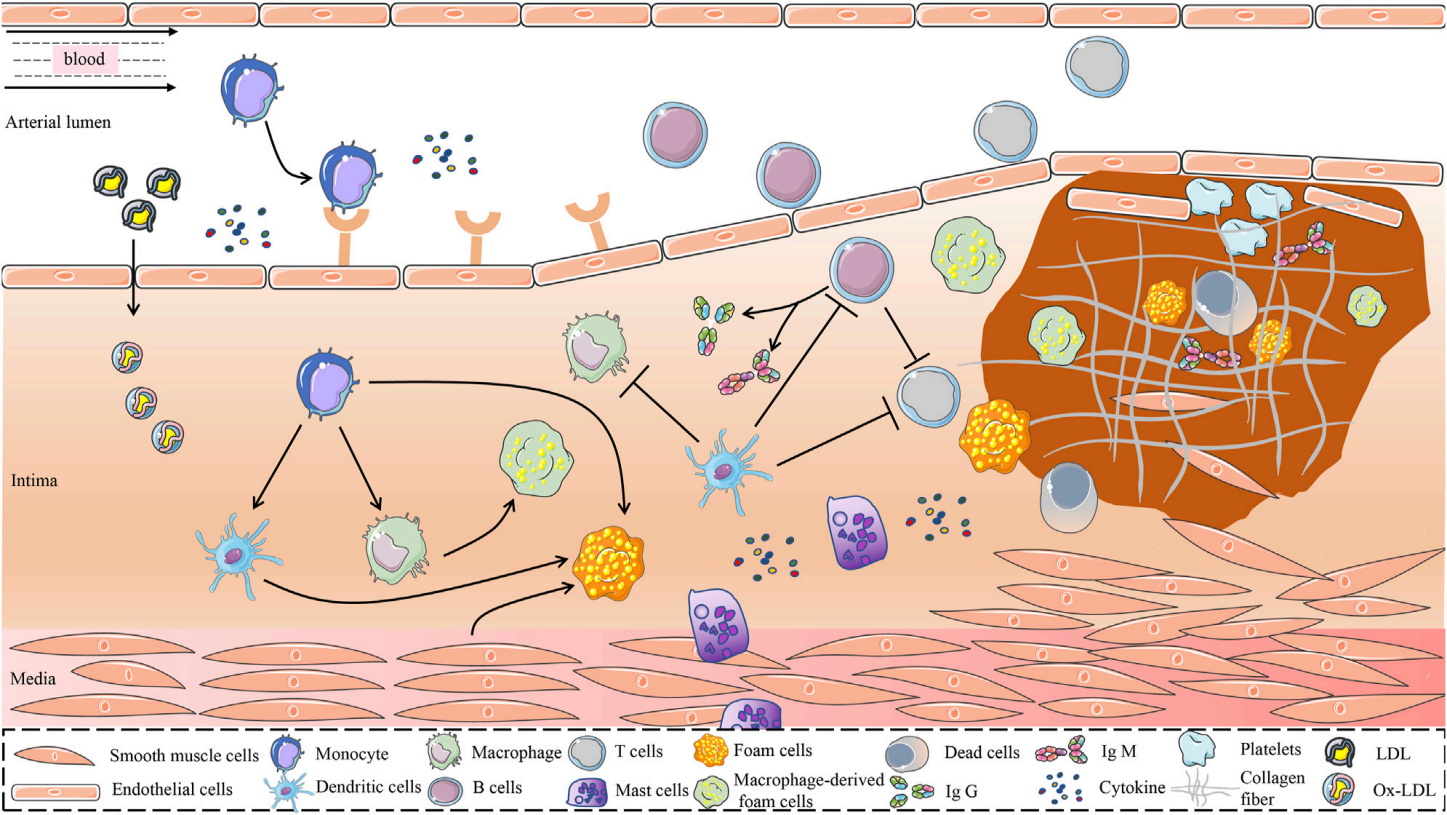
Possible mechanisms for the role of immune cells in the development of AS. The immune system is one of the major regulatory systems in the development and progression of AS. In the early stage of AS, LDL is retained in the intima of the blood vessel and is modified to form a variety of danger-associated molecular patterns mediated by a variety of risk factors, to obtain immunogenicity. Immunogenic LDL activates vascular endothelial cells and attracts immune cells such as monocytes into the vessel wall. Monocytes differentiate dendritic cells and macrophages in response to the stimulation of inflammatory mediators and cytokines. Dendritic cells recognize ox-LDL and present it to immune cells such as macrophages and T cells. Phagocytosis of lipoproteins by monocyte-derived macrophages is an early pathological event in nascent plaque formation. T cells play an important regulatory role in the development of AS. T cells recognize antigens presented by MHC class II molecules on antigen-presenting cells and MHC class I molecules on nuclear cells and activate B cells. In addition, proinflammatory cytokines and anti-inflammatory molecules secreted by T cells play an important role in the development of AS. B cells exert both proatherogenic mechanisms and antiatherogenic effects by secreting IgM and IgG, which are driven by antibodies against specific epitopes of LDL oxidation. Mast cells accelerate LDL penetration and amplify the inflammatory response by releasing histamine, an active mediator, and pro-inflammatory cytokines. Atherosclerotic plaques are formed under the combined action of multiple immune cells, vascular smooth muscle cells, and endothelial cells. AS, atherosclerosis; MHC, Major Histocompatibility Complex; LDL, Low-Density Lipoprotein; ox-LDL, Oxidized Low-Density Lipoprotein.

The current study found that the immune and inflammatory responses have a significant impact on all stages of AS development (Zhu et al., 2018). In the early stages of AS, LDL accumulated in the intima is modified by oxidases, lipolytic enzymes, protein hydrolases, and reactive oxygen species (ROS) to form various damage-associated molecular patterns that acquire immunogenicity and the ability to elicit an immune response (Hansson and Hermansson, 2011). The entry of immunogenic oxidized low-density lipoprotein (ox-LDL) into the intima activates vascular endothelial cells, causing an inflammatory response that recruits chemotactic immune cells into the AS lesion area, such as monocytes and T cells (Abdolmaleki et al., 2019). In the pathological process of AS, the damage of vascular endothelial cells is the beginning of innate immunity, which subsequently activates the mononuclear phagocyte system, leading to the release of large amounts of inflammatory factors (van Dijk et al., 2016). Antigen-presenting cells (APCs), represented by DCs, present a series of processed antigens to T and (or) B lymphocytes to activate adaptive immunity (Roy et al., 2022). As shown in Figure 1.

### 2.1 Monocyte

Endothelial dysfunction is the starting point for the development of AS. Endothelial injury releases adhesion factors and chemokines, such as CC chemokine ligand (CCL) 5, CXC chemokine ligand (CXCL) 1, and P-selectin, which capture circulating monocytes and cause adhesion and aggregation of monocytes (Tacke et al., 2007; Kang et al., 2021). Continuous recruitment of monocytes is necessary for the maintenance and progression of AS plaques (Potteaux et al., 2011).

The study found that monocytes are divided into three main types: classical (CD14^+^CD16^−^ in humans and Ly6Chi in mice), intermediate (CD14^+^CD16^+^ in humans and Ly6C^+^Treml4^+^ in mice), and nonclassical (CD14^−^CD16^+^ in humans and Ly6C^lo^ in mice) (Kim et al., 2020). Classic monocytes are the largest population of monocyte subsets and highly express CXC-chemokine receptor 1, CXC-chemokine receptor 2, and C-C chemokine receptor 2 (Guo et al., 2020). In response to lipopolysaccharide (LPS) stimulation, classic monocytes secrete large amounts of granulocyte colony-stimulating factor and interleukin (IL) 6 (Wong et al., 2011). In addition, classic monocytes also express phagocytosis-related proteins such as CD14, CD64, and CD36, indicating that they have a strong phagocytic capacity (Cros et al., 2010; Zawada et al., 2011). Monocytes that migrate into the plaque differentiate into monocyte-derived macrophages and monocyte-derived DCs. Monocyte-derived macrophages are the main complementary source of inflammatory macrophages in AS plaques. DCs can initiate adaptive immunity by presenting antigens to T cells (Jakubzick et al., 2017). Most non-classical monocytes mainly play a “patrolling” role and do not migrate to target vessels, maintaining the homeostasis of the vascular endothelial environment (Marcovecchio et al., 2017). If the lining of the vessel is damaged or inflamed, the “patrolling” non-classical monocytes can rapidly migrate to the appropriate site. In addition, during the progression of AS, the protective effect of non-classical monocytes is also manifested in removing damaged cells and debris and repairing blood vessels (Thomas et al., 2015). Intermediate monocytes are the transitional cells between mature monocytes and macrophages. Intermediate monocytes have greater transendothelial migration, phagocytosis, and ROS production than classic monocytes (Zawada et al., 2011). Intermediate monocytes can be rapidly recruited to sites of inflammation and secrete large amounts of C-X3-C chemokine receptor 1 and C-C chemokine receptors 2 (Guo et al., 2020), which participate in the inflammatory response.

### 2.2 DCs

As the most powerful APC, DCs play an important role in initiating and amplifying the immune response. Currently, the presence of classical DC (cDC) and plasmacytoid DC (pDC) has been identified in AS lesioned areas in human and AS model mice (Zernecke, 2015). Both cDC and pDC have been detected in both mouse and human AS lesions. Several cDC isoforms have been detected in arterial tissue, including CD103^+^ DC, CD11b^+^ DC, and CCL17^+^ DC (Gardner et al., 2020).

DCs act as central stimulators of the immune response, exerting a bidirectional pro- and anti-AS effect. Activated DCs secrete cytokines and chemokines that exert long-range effects. Such as DCs can recruit circulating leukocytes to vascular areas (Bellini et al., 2022) and regulate the differentiation of T cells into a variety of T effectors, including T helper (Th) type 1 cells, Th1, Th2, Th17, and Tregs (Markin et al., 2023). They can also activate cytotoxic T lymphocyte and B cells and polarize macrophages, leading to immune destruction of the vessel wall and induction of AS (Kloc et al., 2022). As specialized APCs, cDCs recognize antigens in the vessel wall and present them to antigen-specific naïve T cells in the lymph nodes or spleen. In addition, DCs can directly ingest lipids to form foam cells (Haka et al., 2015).

### 2.3 Macrophages

The origin of macrophages in AS plaques can be divided into two categories: resident macrophages derived from embryonic progenitor cells and peripheral monocyte-derived macrophages (Denardo and Ruffell, 2019; Williams et al., 2020), with monocyte-derived macrophages predominating (Shapouri-Moghaddam et al., 2018). When AS occurs, resident macrophages are replenished and self-renewed by recruiting mature peripheral monocytes into the tissue. In addition, with the development of AS, replenishment of resident macrophages within the AS plaques can also be achieved by the proliferation of macrophages *in situ* (Farahi et al., 2021).

Macrophages, as the main immune effector cells, are involved in all stages of AS, from the occurrence to the formation of a necrotic core (Tabas and Bornfeldt, 2016). The uptake of lipoproteins by macrophages via scavenger receptors leads to the formation of lipid-rich foam cells (Chistiakov et al., 2019). This exacerbates the development of lipid streaks. Recent studies have found that cells with foam characteristics are enriched in a large number of anti-inflammatory genes and lipid processing genes, and relatively few inflammatory genes, suggesting that the formation of foam cells may drive the regression of inflammation in the lesion area (Kim et al., 2018; Depuydt et al., 2020). The excessive accumulation of lipoproteins and aggravation of oxidative stress leads to metabolic disorders of macrophages, which trigger a series of inflammatory responses and induce the infiltration of immune cells in AS plaques (Kojima et al., 2017).

Macrophages are classically characterized by diversity and plasticity (Mantovani et al., 2013). Traditionally, macrophages are usually dichotomized as M1 and M2, but this does not encompass the multiple functions performed by macrophages (Atri et al., 2018; Moreira et al., 2020). In addition, macrophage phenotypes include Mox, HA-mac, Mhem, and M (Hb) types (Boyle et al., 2009; Marques et al., 2016; Liberale et al., 2017). Current studies mainly analyze the role of M1 and M2 macrophages in AS. Classically activated M1 macrophages are pro-inflammatory cells that are induced to polarize by granulocyte-macrophage colony-stimulating factor, LPS, tumor necrosis factor-α (TNF-α), and ox-LDL (Shapouri-Moghaddam et al., 2018; Eshghjoo et al., 2022). M1 macrophages express a series of pro-inflammatory factors, including TNF-α, IL-1β, IL-12, IL-23, chemokine ligands CXCL9, CXCL10, and CXCL11 (Yunna et al., 2020), which play an important role in the initiation of inflammation. It promotes the progression of AS and affects the stability of plaques. In contrast, alternative activated M2 macrophages are anti-inflammatory cells that can be polarized by L-4, IL-13, CCL17, IL-10, transforming growth factor-β (TGF-β), and immune complexes (Gordon and Martinez, 2010). M2 macrophages typically express high levels of IL-10 and low levels of IL-12, which effectively inhibit the inflammatory response and stabilize AS plaques (De Paoli et al., 2014). M2 macrophages are predominantly enriched in stable plaques. As the stability of plaques decreased, M1 macrophages gradually replace M2 macrophages in dominance (Stoger et al., 2012; Amengual and Barrett, 2019). The mechanism of macrophages in AS is complex. An in-depth exploration of the specific roles of macrophage subsets in AS is essential to understand their role in the progression of AS and developing novel therapeutic drugs.

### 2.4 Mast cells

Mast cells, as important effector cells of the innate immune response, are involved in almost all stages from the occurrence of AS to plaque rupture, and their number increases with the progress of AS (Kovanen and Bot, 2017). Histamine, the major active mediator of mast cells, can promote endothelial histamine H1 receptor expression, increase the permeability of aortic endothelium to circulating LDL, and accelerate the penetration of LDL particles into the susceptible intima (Rozenberg et al., 2010). Previous studies have shown that there is an accumulation of mast cells in human AS plaques, and the number of activated mast cells is significantly increased around the diseased vessels (Atkinson et al., 1994). Mast cells amplify the inflammatory response by releasing the pro-inflammatory cytokines IL-6 and interferon-γ (IFN-γ) (Sun et al., 2007) and increase plaque instability by releasing the chemokines CXCL1 and CXCL12 to induce neutrophil recruitment to the site of AS inflammation (Wezel et al., 2015). Generally, calcification of AS plaques is regarded as a protective factor against AS. Mast cell activation was negatively correlated with the macrocalcification of AS plaques and positively correlated with plaque fragility parameters (Skenteris et al., 2023). Stress-induced activation of mast cells has been found to cause intraplaque hemorrhage and increase plaque instability in ApoE^−/−^ mice (Lagraauw et al., 2019). In addition, mast cells also induce apoptosis of smooth muscle cells leading to the rupture of atherosclerotic plaques (Leskinen et al., 2003). In the reprogramming of smooth muscle cells, mast cells stimulate smooth muscle cell calcification and induce their differentiation into a proinflammatory, osteochondrocyte-like phenotype (Skenteris et al., 2023). Under hyperlipidemic conditions, mast cells highly express major histocompatibility complex class II molecules, specifically activating the accumulation of CD4^+^ T cells within AS plaques by acting as APC (Kritikou et al., 2019).

### 2.5 T cells

After AS-related antigens are recognized by DCs and macrophages, MHC class molecules on the surface of APCs activate naive CD4^+^ and CD8^+^ T cells *in vivo* by binding to T cell surface receptors (Nilsson and Hansson, 2020). Subsequently, CD4^+^ and CD8^+^ T cells differentiate into various subsets in response to stimulation by different cytokines. For example, CD4^+^ T cells can differentiate into helper T cells and regulatory T cells, and CD8^+^ T cells can differentiate into cytotoxic T lymphocytes. T cell subsets have shown diverse roles in the complex AS microenvironment.

In different stages of AS, CD4^+^ T cell subsets play an immune-activating or immunosuppressive role and can assist in the activation of B cells to produce antibodies (Saigusa et al., 2020). It is generally accepted that pro-inflammatory CD4^+^ effector T cells are dominant during the developmental phase of AS, whereas Treg cells are dominant during the pre- or stabilization phase of AS (Buono et al., 2005; Ait-Oufella et al., 2006). T helper cell plasticity provides a challenge to our understanding of the role of Th cells in AS. Studies have found that in the late stage of AS, there are a large number of Th1 cells, which can not only enhance antigen presentation but also secrete IFN-γ, TNF, and IL to promote inflammatory response (Wolf and Ley, 2019). IFN-γ activated macrophages to pro-inflammatory polarization and promoted VSMC proliferation (Saigusa et al., 2020). In addition, IFN-γ can induce the formation of foam cells in the progressive stage of AS (Yu et al., 2015). TNF, which is expressed by a variety of effector T cells, promotes the migration of circulating leukocytes to sites of vascular endothelial injury, thereby contributing to the development of AS (Biros et al., 2022).

The role of Th2 cells in AS remains controversial, but the most convincing evidence suggests that Th2 cells primarily play an anti-AS role (Binder et al., 2004; Gronberg et al., 2017). Activated Th2 cells release IL-4, IL-5 and IL-13. IL-4 inhibits Th1 responses and is considered a protective factor against AS in ApoE^−/−^ mice (Davenport and Tipping, 2003). However, exogenous IL-4 did not inhibit AS in ApoE^−/−^ mice (King et al., 2007). This has led to a debate on the role of IL-4 for AS. The protective effects of IL-5 and IL-13 on AS have been relatively clear. A negative correlation between IL-5 and intima-media thickness was found in female common carotid segments, suggesting that IL-5 may be part of the protective mechanism in the early of AS (Silveira et al., 2015). Mechanistically, IL-5 stimulates B1 cells to produce IgM to prevent the uptake of ox-LDL by macrophages, thereby inhibiting the formation of necrotic cores (Munjal and Khandia, 2020). In addition, IL-5 upregulates the expression of ATP-binding cassette transporter A1 (ABCA1) by regulating the miR-211/JAK2/STAT3 signaling pathway to promote cholesterol efflux (Chen et al., 2020). In a retrospective study, lower levels of IL-13 were found to be associated with higher carotid intima-media thickness values (Boccardi et al., 2020). This suggests that IL-13 deficiency accelerates the progression of AS. IL-13 inhibits the recruitment of vascular cell adhesion molecule 1 (VCAM-1)-dependent monocytes to suppress the inflammatory response and promotes the formation of stable plaques in AS by increasing collagen content in the lesion area and modulating the macrophage phenotype (Cardilo-Reis et al., 2012).

The role of IL-17 in AS is still debated. Th17 cells secrete IL-17, IL-21, and IL-22, among which IL-17 is the signature cytokine of Th17 cells. Infiltrating Th17 cells in the arteries of patients with coronary atherosclerosis secreted large amounts of IL-17, which acted synergistically with IFN-γ to induce vascular smooth muscle cells (VSMCs) to participate in the inflammatory response (Eid et al., 2009). As a member of the IL-17 family, IL-17A promotes inflammatory responses by facilitating the recruitment of monocytes and macrophages to the AS region (Smith et al., 2010). In addition, IL-17A stimulates ox-LDL-induced foam cell formation by promoting macrophage expression of lectin-like oxidized low-density lipoprotein receptor 1 (Shiotsugu et al., 2019). The use of neutralizing antibodies against IL-17A can significantly inhibit the production of proinflammatory cytokines and chemokines (Butcher et al., 2016). However, studies in ApoE^−/−^ IL-17A^−/−^ mice found that IL-17A deficiency accelerated the progression of AS and promoted the formation of unstable plaques (Danzaki et al., 2012). In the hypercholesterolemic environment, promoting IL-17 expression contributes to plaque stabilization (Brauner et al., 2018). The complex target role of IL-17 provides a direction for the study of the immune mechanism of the occurrence and development of AS.

At present, it is generally believed that Treg cells play a protective role in AS (He et al., 2020). Treg cells secrete immunosuppressive cytokines such as IL-10 and TGF-β to inhibit the excessive inflammatory response, and enhance immune tolerance and immune balance through cell contact-dependent mechanism and tissue repair (Spitz et al., 2016; Sakaguchi et al., 2020). Adoptive transfer of Treg cells into the aorta of ApoE^−/−^ mice significantly reduced macrophage infiltration and reduced AS plaque area in the aorta (Gao et al., 2020). Depletion of Treg cells in AS model mice using a CD25 monoclonal antibody affected the alternative activation of macrophages, efferocytosis, upregulation of pro-lipid regression mediators, inhibition of Th1 responses, and blocked the remodeling and contraction of AS plaques (Sharma et al., 2020). The above suggests that Treg cells play an important protective role in AS. However, the mechanisms by which Treg cells interact with various cells associated with AS are still not fully understood, and this gap needs to be filled.

Several studies (Cochain et al., 2018; Zernecke et al., 2020) have confirmed that CD8^+^ T cells are enriched and infiltrated in AS plaques in humans and mice. CD8^+^ T cells in plaques are highly activated compared to T cells in circulation (Grivel et al., 2011). CD8^+^ T cells in lesioned areas of AS in Ldlr^−/−^ mice fed with a high-fat diet highly expressed IFN-γ, TNF-α, and IL-12, and antibody-mediated depletion of CD8^+^ T cells inhibited the development of AS (Cochain et al., 2015). However, in ApoE^−/−^ mice lacking antigen processing-associated transporter protein-1, a significant reduction in CD8^+^ T cell levels did not affect the progression of AS (Kolbus et al., 2012). Notably, the increased infiltration of CD4^+^ T cells in AS lesions compensated for the absence of CD8^+^ T cells, which may mask the impact of CD8^+^ T cell deficiency on AS progression. In addition, CD8^+^ T cells can differentiate into subpopulations with specific cytokine profiles. CD8^+^ Tc17 T cells do not affect the development of early AS although they accumulate in the lesioned areas of mice with AS (van Duijn et al., 2021). In contrast, CD8^+^ IFN-γ^+^ T cells mediate the upregulation of immune responses and secretion of pro-inflammatory cytokines during the progressive phase of AS, exacerbating AS load and promoting the inflammatory response (Li et al., 2023). On the one hand, inflammatory cytokines produced by CD8^+^ T cells exacerbate the inflammatory response and drive the progression and instability of atherosclerotic lesions. On the other hand, cytotoxic activity against APCs and the presence of regulatory CD8^+^ T cell subsets suppress immunity and can limit AS (van Duijn et al., 2018).

### 2.6 B Cells

Mouse B cells can be broadly divided into B1 and B2 cells. Due to the differences in the surface markers and immune cell responses of human B cells and mice, the B cell subtypes and functions of human B cells are different from those of mice. However, the function of human B-cell subsets can be inferred from the function of mouse B-cell subsets in AS. Unlike T cells, only a small number of B cells can be found in localized lesions of AS (Poznyak et al., 2021).

At present, there is a large amount of evidence that B cell subsets play a crucial role in AS (Sage et al., 2019). B1 cells differentiated into B1a and B1b cells, and B2 cells differentiated into follicular B (FO B) cells and marginal zone B (MZ B) cells. In addition, the B cell subpopulation includes innate response activator B (IRA B) cells and Bregs. Among them, B1 cells and MZ B cells exhibit a protective role in AS (Tsiantoulas et al., 2015). In contrast to IRA B and FO B cells, which play a role in promoting AS formation (Tsiantoulas et al., 2015). Studies have found that the deficiency of B1 cells aggravates AS, while the adoptive transfer of B1 cells has an inhibitory effect on AS (Kyaw et al., 2011). The mechanism is related to the secretion and the accumulation of IgM, which inactivates oxidation-specific epitopes of LDL (Kyaw et al., 2011). In contrast, B2 cells secrete IgG and IgE that promote AS (Moriya, 2019), but this remains controversial. B2 cells differentiate into memory B cells and plasma cells, and the increase of plasma cells is associated with AS (Nutt et al., 2015). Studies have found that MZ B cells delay the progression of AS by secreting IgM and inhibiting T follicular helper cells (Nus et al., 2017). The decrease of FO B cells and the increase of Berg cells significantly attenuated AS (Douna et al., 2020). The T cell-mediated differentiation of FO B cells into plasma cells and secretion of pathogenic IgG is an important mechanism for the pro-AS effect of FO B cells (Tay et al., 2018). In addition, the pro-AS effect of FO B cells is highly dependent on their expression of MHC II, CD40, and Blimp-1 (Tay et al., 2018). Granulocyte-macrophage colony-stimulating factor-producing IRA B cells alter adaptive immune processes and shift the leukocyte response toward a Th1-associated milieu that aggravates AS (Hilgendorf et al., 2014). Breg cells have not been studied in depth yet. Breg cells can inhibit AS by secreting IL-10 (Ponnuswamy et al., 2017). Interestingly, in Ldlr^−/−^ mice with B-cell-specific deficiency in IL-10, B-cell-derived IL-10 does not alter AS in mice (Sage et al., 2015). The research and exploration of the specific role of B cells in AS is still in its infancy, and it is necessary to continue to study the complex regulatory role of B cells and their cytokines in the environment of AS.

## 3 Overview of the GM

GM is mainly colonized in the gastrointestinal tract and includes bacteria such as probiotics, neutrophils, and pathogens, as well as microorganisms such as viruses that coexist with the host, 99% of which are bacteria (Neish, 2009; Jandhyala et al., 2015). The adult intestine is inhabited by at least 1,000 microorganisms, with the number increasing from the esophagus, stomach, duodenum, and small intestine to the colon (Clemente et al., 2012). The number of GM in the human body far exceeds the number of cells in the human body (Van Treuren and Dodd, 2020). The GM genome contains at least more than 9 million genes and is known as the second genome in the body (Li et al., 2014). With the development of high-throughput sequencing technologies such as macroeconomics, 16SRNA, and metabolomics, we have a more precise understanding of the classification and function of GM. The mystery of GM has been gradually unveiled. There is a delicate balance between tens of thousands of GM and their host.

The GM is inter-constrained and interdependent to maintain the balance of the intestinal environment. The balance of the microbiota is an important guarantee for the normal physiological function of the organism. The homeostasis of the bacterial microbiota is dominated by a dynamic equilibrium with interactions among its stabilizing factors, including the immune barrier, host intestinal mucosa, intestinal microecology, and metabolites (Zhou et al., 2021; Shen et al., 2022). When the balance of microbiota in the body is imbalanced, it can lead to several systemic diseases. GM is now recognized as an important factor in keeping the body healthy and influencing the development of disease (Perler et al., 2023). Therefore, effective maintenance of the dynamic balance of the GM is the key to ensuring the health of the human body and the healing of diseases.

## 4 GM, their metabolites, and AS

Studies have shown that GM and its metabolites may prevent or promote the progression of AS by modulating host metabolism and inflammatory responses (Brandsma et al., 2019). AS plaque itself is a microbial environment, which contains *Streptococcus*, *Pseudomonas*, *Klebsiella*, and *Chlamydia pneumoniae* (Koren et al., 2011; Lanter et al., 2014). A systematic review and Meta-analysis showed that the abundance of GM was reduced in patients with coronary artery disease and that alterations in GM composition were associated with qualitative and quantitative changes in bacterial metabolites (Choroszy et al., 2022). Compared to healthy controls, the relative abundance of GM was more significantly altered in patients with symptomatic AS, such as the higher abundance of *Collinsella genus*, *Enterobacteriaceae*, *Streptococcaceae*, and *Klebsiella* spp., and lower abundance of SCFAs-producing *Eubacterium*, *Roseobacter,* and *Rumenococcus spp* (Jie et al., 2017; Liu et al., 2019; Zhao et al., 2020). *Fusobacterium* and *Proteus* in the GM were found to be associated with carotid plaque, while beneficial butyrate producer *Odoribacter* was found to be negatively associated with plaque in people at high risk of HIV infection (Wang et al., 2022). However, another study found no significant differences in bacterial DNA amounts or microbial composition between plaques in patients with symptomatic and asymptomatic AS, or between different plaque regions (Lindskog et al., 2017). This suggests that there are other more critical factors in determining plaque susceptibility, which need further study. Pathogenic bacteria in the GM can promote the formation of plaques on the vascular wall by directly infecting the vascular wall or by causing autoimmune inflammatory responses through molecular mimicry (Epstein et al., 2000).

However, interventions with antibiotics applied to eliminate microbiota in plaques were found not to reduce the incidence of cardiovascular events (Song et al., 2008). The reasons for this result cannot be excluded to be related to the fact that the duration of the antibiotic intervention was too short and that the antibiotics applied did not target all microbiota (Jonsson and Backhed, 2017).

**FIGURE 2:**
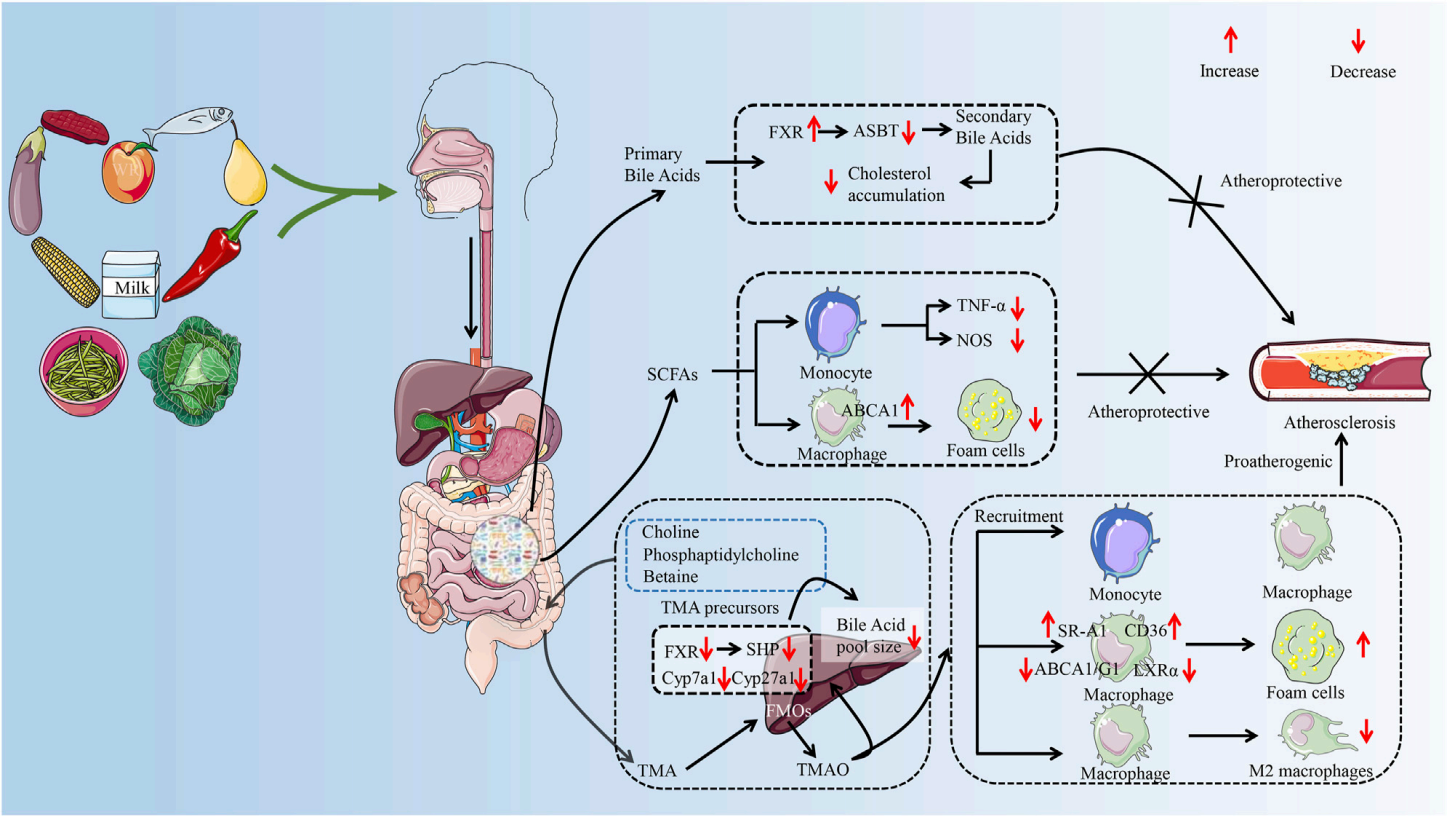
The possible mechanism of GM metabolites (TMAO, SFCAs, secondary bile acids) to exert pro-AS or anti-AS activities by regulating immune cells. The GM helps the body digest food, breaking down nondigestible cellulose and other complex carbohydrates. After entering the gut, food is metabolized by GM to primary bile acids, trimethylamine, and SCFAs. Trimethylamine is oxidized to trimethylamine oxide in the liver by FMO 3. In the progression of AS, TMAO can promote the recruitment of monocytes and macrophages and accelerate the inflammatory response. TMAO disrupted cholesterol homeostasis in macrophages by affecting the expression of ABCA 1, ABCG 1, and LXRα. It promotes the formation of macrophage-derived foam cells and accelerates the formation of atherosclerotic plaques. In addition, TMAO also prolonged the inflammatory response phase by inhibiting the polarization of the M2 phenotype of macrophages. SCFAs are considered to be protective factors for delaying the progression of AS. SCFAs can reduce foam cell formation not only by inhibiting monocyte-induced inflammatory response but also by promoting ABCA 1-mediated cholesterol efflux from macrophages. Secondary bile acids play a role in inhibiting AS by inhibiting cholesterol accumulation. GM, gut microbiota; AS, atherosclerosis; TMA, trimethylamine; SCFAs, short-chain fatty acids; FMO 3, flavin-containing monooxygenase 3; ABCA 1, ATP-binding cassette proteins A 1; ABCG 1, ATP-binding cassette proteins G 1; LXRα, liver X receptor α; FXR, farnesoid X receptor; ASBT, Apical Sodium-dependent Bile Acid Transporter; TNF-α, tumor necrosis factor-α; NOS, nitric oxide synthase; SHP, small heterodimer partner. (Some material taken from *Servier Medical Art* and *freepik*.)

A synthesis of the current reports reveals that studies mainly focus on the effect of GM metabolites on AS. Such as SCFAs, secondary bile acids, and TMAO are an important part of the development of AS (Brown and Hazen, 2015; Jonsson and Backhed, 2017). The role of GM metabolites in AS has received widespread attention (Brown and Hazen, 2018), which provides a new perspective on the treatment of AS from the perspective of GM. The effect of different metabolites of the GM on AS varies. Taking TMAO and SCFAs as examples, it was reported that elevated plasma TMAO levels promote the development of AS and are associated with the risk of cardiovascular disease (Qi et al., 2018; El et al., 2023). However, SCFAs exhibit anti-AS properties (Bultman, 2018). Therefore, the effect of GM on AS needs to be discussed separately depending on the metabolite (as shown in Figure 2). This article focuses on a review of the mechanisms by which metabolites of the GM (SCFAs, secondary bile acids, and TMAO) regulate immune cells to influence the progression of AS.

## 5 Crosstalk between GM, its metabolites, and immune inflammatory response in AS

### 5.1 Bacteria and bacterial components

Under normal circumstances, beneficial and pathogenic bacteria maintain a homeostatic balance in the gut ecology and compete for nutrients to act as antagonists against foreign pathogens and prevent pathogenic bacteria from colonizing (Coyne and Comstock, 2019). Changes in the composition of GM directly affect the changes in microbiota-associated products and influence the intestinal barrier structure through various pathways. For example, when the abundance of *Gram-negative bacilli* increases, the LPS, the main virulence factor on the surface of the bacterial membrane, increases accordingly. Disruption of the intestinal barrier and increased permeability lead to the entry of bacteria and bacterial membrane components represented by LPS into the circulating blood (Gorabi et al., 2022). After entering the body, LPS is recognized by Toll-like receptors (TLR) and activates NF-κB to cause an immune response, leading to the aggregation of monocytes and macrophages, increased release of coagulation factors, inflammatory factors, and oxygen free radicals, and further worsening the situation of increased intestinal permeability, eventually leading to more LPS invasion of the body, forming a vicious cycle (Abdollahi et al., 2022). In AS plaques, macrophages activated by LPS acquire hyperinflammatory tissue destruction features and amplify Th1 and Th17 cell-mediated responses (Kuznetsova et al., 2020). In addition, LPS promotes the lesion of AS by enhancing the inflammatory properties of senescence and senescence-associated secretory phenotype in specific vascular cells (Suzuki et al., 2022).

In contrast, probiotics such as *Bifidobacterium* break down indigestible dietary fiber and resistant starch into SCFAs, and enhance the intestinal barrier through adenosine monophosphate-activated protein kinase (AMPK) (Peng et al., 2009; Parada et al., 2019). *Bacteroides vulgatus* and *Bacteroides dorei* significantly improve endotoxemia and suppress pro-inflammatory immune responses by reducing LPS production, thereby delaying the formation of AS (Yoshida et al., 2018). Recent studies have found that *Lactobacillus* strains enhance mucosal immunity by increasing secretory IgA in mice (Katiraei et al., 2020). In addition, *Bifidobacterium* reduces choline-induced plasma TMAO production by modulating the GM in mice, thereby inhibiting the progression of AS (Qiu et al., 2017; Wang et al., 2022).

In AS, the changes in GM in each phylum were similar to a large extent. At the phylum level, GM in AS patients is mainly characterized by an increased relative abundance of *Firmicutes*, decreased relative abundance of *Bacteroidetes*, and increased *Firmicutes*/*Bacteroidetes* ratio (Verhaar et al., 2020). At the genus level, it was manifested as a decrease in the relative abundance of probiotics such as *Bifidobacterium* and an increase in the relative abundance of pathogenic bacteria such as *Klebsiella* and *Escherichia*. Specifically, the relative abundance of *Ruminococcus*, *Lactobacillaceae*, and *Streptococcus* in *Firmicutes* increased, and the relative abundance of *Aerococcaceae*, *Eubacterium*, and *Faecalibacterium* decreased (van den Munckhof et al., 2018; Hashizume-Takizawa et al., 2019). The relative abundance of *Bacteroides*, *Prevotella*, *Bacteroide*s, and *Prevotella* decreased in *Bacteroidetes* (van den Munckhof et al., 2018). In addition, the relative abundance of *Collinsella* and *Eggerthella* in *Actinobacteria* increased, and the levels of *Bifidobacterium* and *Bifidobacterium genera* decreased (Chen et al., 2016; Zhao et al., 2020). The relative abundance of *Enterobacter aerogenes*, *Klebsiella*, and *Escherichia* in *Proteobacteria* decreased (Lanter et al., 2014; Qiu et al., 2017). Studies have shown that *Firmicutes* and *Proteobacteria* are the majority of bacterial DNA detected in atherosclerotic plaques, which are associated with plaque formation and instability (Calandrini et al., 2014; Chen et al., 2016). It should be pointed out that the relationship between GM and AS is still under continuous research, and further in-depth scientific exploration is needed to clarify the mechanism and effect of GM regulation of AS and to clarify the close relationship between GM and AS.

### 5.2 Metabolites of GM

#### 5.2.1 TMAO

Trimethylamine (TMA) is a low-boiling, nitrogen-containing small-molecule compound (Goel et al., 2016). Dietary precursors such as choline, phosphatidylcholine, and L-carnitine, which are rich in meat, dairy products, eggs, and fish, are produced TMA by TMA lyase of GM (Simo and Garcia-Canas, 2020; Luo et al., 2022). After absorption from the intestine, TMA enters the liver via the portal vein and is oxidized to TMAO by the flavin⁃containing monooxygenase (FMO) family, especially FMO3 (Gatarek and Kaluzna-Czaplinska, 2021). *Nature* reported in 2011 that TMAO independently predicted the risk of peripheral artery disease, coronary artery disease, and myocardial infarction (Wang et al., 2011). Since then, the relationship between TMAO and AS has attracted the attention of researchers (Ascher and Reinhardt, 2018).

A prospective nested case-control study found that long-term increases in TMAO were associated with a high risk of coronary heart disease and that long-term repeat assessment of TMAO improved the identification of people at high risk of coronary heart disease (Heianza et al., 2020). In addition, TMAO can also be used as a prognostic indicator of cardiovascular events in patients with acute coronary syndromes and stroke (Li et al., 2017; Tan et al., 2019). Plasma TMAO levels were higher in patients with healed plaques than in patients with ST-segment elevation myocardial infarction with unhealed plaques (Li et al., 2021). TMAO has been considered a risk marker for cardiovascular disease (Jaworska et al., 2019). However, a study with people aged 35–55 years showed no significant association between TMAO and the development of AS (Meyer et al., 2016). It is speculated that the reason for this result may be related to the population involved in this study, which was selected from a relatively young population, and that most of the young patients were in the early stages of AS progression, so the effect of TMAO on AS has not yet appeared. In addition, young people have better kidney function and TMAO can be excreted from the body in time. It is worth mentioning that another study found that TMAO exacerbated AS in a low-fat diet but not in a high-fat diet state (Yu et al., 2020). In summary, some progress has been made on the mechanism of TMAO in promoting AS. TMAO was found to induce the development and progression of AS in various ways, including by promoting inflammatory responses and affecting cholesterol metabolism (Zheng and He, 2022).

##### 5.2.1.1 Promotes the recruitment of monocytes and macrophages

AS is commonly defined as a chronic inflammatory disease caused by inflammatory damage to the vascular endothelium, and damage to the vascular endothelium is the induced event of AS (Hooglugt et al., 2022). TAMO accelerates the progression of AS by damaging the vascular endothelium to recruit monocytes and macrophages to promote an inflammatory response (Seldin et al., 2016). It was found that TMAO activates the NF-κB signaling pathway by activating protein kinase C, promoting the expression of cell adhesion factor-1 (such as VCAM-1), and increasing the adhesion of monocytes, thereby accelerating endothelial dysfunction (Ma et al., 2017). In addition, TMAO can also promote inflammatory responses by enhancing MAPK/ERK activity to activate the NF-κB signaling pathway (Yang et al., 2019a).

In a study involving 5 HIV-infected and 520 HIV-uninfected subjects (217 incident plaque cases), plasma TMAO was found to be positively correlated with serum sCD14 and sCD163 levels (Shan et al., 2018). sCD14 and sCD163 are biomarkers of monocyte activation and macrophage inflammation, suggesting that TMAO is closely associated with monocyte- and macrophage-mediated inflammatory responses. In the aorta of choline-fed mice, the expression of monocyte chemotactic protein 1, macrophage inflammatory protein 2, TNF-α, ICAM-1, E-selectin, and VCAM-1 was significantly enhanced, and the macrophage marker CD68 was also highly expressed, which enhanced the recruitment of leukocytes such as monocytes and macrophages to endothelial cells (Seldin et al., 2016). The adhesion of monocytes to endothelial cells may be related to the activation of NLRP3 inflammasome by TMAO through the SIRT3-SOD3-mtROS signaling pathway (Chen et al., 2017). Previous studies have confirmed that Proline/serine-rich coiled-coil protein 1 (PSRC1) has important effects on the immune system (Tonjes et al., 2018). PSRC1 deficiency leads to a significant increase in TMAO levels, which in turn promotes infiltration of F4/80^+^ macrophages in AS plaques (Luo et al., 2022). At the same time, this process was accompanied by an upregulation of mRNA levels of pro-inflammatory cytokines (including IL-6, IL-10, TNF-α, and IL-17A), in contrast to a significant decrease in the expression levels of anti-inflammatory cytokines (including IL-10, IL-4, IL-13, Ym-1, and Ym-2) (Luo et al., 2022). *In vitro*, TMAO significantly increased ox-LDL-induced migration of macrophages, further confirming that TMAO enhanced macrophage recruitment.

##### 5.2.1.2 Disturbance of macrophage cholesterol homeostasis and promotion of macrophage-derived foam cell formation

Cholesteryl ester-rich foam cells are an important component of AS plaques. Foam cells in AS plaques are mainly derived from macrophages and smooth muscle cells (Wang et al., 2022). Choline promotes cholesterol accumulation in the macrophages of ApoE^−/−^ mice, which in turn accelerates the formation of macrophage-derived foam cells and ultimately exacerbates the development of AS (Wang et al., 2015). High-density lipoprotein promotes cholesterol efflux through ATP-binding cassette proteins A 1 (ABCA1), ATP-binding cassette proteins G 1, and scavenger receptor class B type I (SR-B1) protein transporters to promote cholesterol efflux and thus prevent cholesterol deposition, which is the first step of reverse cholesterol transport (RCT) (Ouimet et al., 2019). RCT transfers excess cholesterol from cells to the liver, where it is excreted from the body through transfer to bile as free cholesterol or through feces after conversion to bile acids, a process that is thought to inhibit AS (Getz and Reardon, 2018). RCT was significantly inhibited in macrophages of mice fed TMAO (Koeth et al., 2014). RCT disorders lead to the subcutaneous deposition of LDL in the arteries, which are oxidized to ox-LDL by ROS. Ox-LDL stimulates endothelial cells to secrete large amounts of inflammatory mediators, which drives inflammatory cells to infiltrate into the subendothelium leading to chronic inflammation and ultimately causing AS (Lu et al., 2022).

Knockout of FMO3 in mice stimulates liver X receptor α to induce macrophage-mediated RCT, which in turn inhibits intestinal cholesterol absorption and improves cholesterol homeostasis (Warrier et al., 2015). TMAO elevates serum cholesterol levels by decreasing the expression of SR-B1, Abcg5/g8, and Cyp7a1, which in turn promotes the development of AS (Zhu et al., 2016; Canyelles et al., 2018). The scavenger receptor A 1 (SR-A1) and CD36 are not subject to negative feedback regulation from intracellular cholesterol and can sustain cholesterol uptake to induce macrophage transformation into foam cells (Okazaki et al., 2015; Xia et al., 2020). TMAO mediates the phagocytosis of large amounts of cholesterol by macrophages by up-regulating the expression of CD36 and SR-A1 (Wang et al., 2011), and significantly enhances the intracellular load of cholesterol in macrophages. TMAO inhibits cholesterol transport by downregulating the expression of ABCA1 in macrophages to promote lipid accumulation (Mohammadi et al., 2016). In addition, the CD36/MAPK/JNK pathway may play an important role in TMAO-induced lipid accumulation as well as the formation of foam cells (Geng et al., 2018; Shi et al., 2021).

The human body converts about 0.5 g of cholesterol into bile acids every day, which is almost half of the cholesterol consumed by the body every day (Li and Dawson, 2019). The synthesis of bile acids is the main pathway of cholesterol catabolism (Hu et al., 2022). Bile acid metabolism has an important role in cholesterol transport, and the upregulation of bile acid synthase expression helps to enlarge the bile acid pool to increase cholesterol transport. The downregulation of bile acid synthase (Cyp7a1 and Cyp27a1) and bile acid transport protein expression (Oatp1, Oatp4, Mrp2, and Ntcp) inhibits the transportation of cholesterol, resulting in intracellular accumulation of cholesterol and the formation of foam cells, which leads to the development of AS (Koeth et al., 2013; Xu et al., 2021). The decrease in bile acid synthesis leads to the accumulation of large amounts of cholesterol in the body (Bove et al., 2004). TMAO further inhibits bile acid synthesis by activating the nuclear receptor farnesoid X receptor (FXR) and small heterodimer partner (SHP) and inhibiting the expression of Cyp7a1 (Ding et al., 2018), thereby accelerating the formation of aortic lesions in mice. In conclusion, the above studies suggest that TMAO affects cholesterol metabolism through multiple pathways, promoting lipid accumulation and the formation of macrophage-derived foam cells.

##### 5.2.1.3 Other

Macrophage polarization is an activity in which macrophages change phenotype and show differential functions under the influence of complex plaque microenvironments to adapt to the adjustment of the surrounding environment. In the plaque microenvironment, macrophages are stimulated by multiple signals simultaneously and polarized into different subtypes. M1 macrophages are mainly pro-inflammatory and accelerate the development of AS (Stoger et al., 2012). In contrast, M2 macrophages are mainly anti-inflammatory and promote tissue repair, which can inhibit the development of lesions to some extent (Shapouri-Moghaddam et al., 2018). The imbalance between the proportion of M1 and M2 macrophages affects the development of AS. A study conducted in a C57/BL6 mouse model of unstable carotid plaques found that TMAO inhibited the cytostatic effect of macrophages and polarization to the M2 phenotype, which reduced the stability of AS plaques (Shi et al., 2021). However, it was found that the downregulation of TMAO levels had no significant effect on the polarization of M1 macrophages (Shi et al., 2021). In addition, TMAO induces endoplasmic reticulum stress involved in foam cell formation and abnormal macrophage activation by promoting the expression of heat shock proteins and glucose-regulated proteins, such as HSP70, HSP60, GRP94, and GRP78 (Yang et al., 2019b).

Oxidative stress refers to the imbalance between the production of intracellular oxides and the antioxidant effect when the body is stimulated by various risk factors, which ultimately damage the biological system of the organism (Koutsaliaris et al., 2022). Elevated levels of TMAO inhibit the expression of Sirtuin 1 and thus promote oxidative stress (Ke et al., 2018). TMAO activates oxidative stress and increases the levels of interleukin-1β and interleukin-18 by inhibiting the activation of endothelial NOS and the expression of nitric oxide (Sun et al., 2016). NLRP3 inflammatory vesicle activation and oxidative stress were significantly attenuated in carotid ligation mice fed TMAO inhibitors, which inhibited vascular remodeling (Chen et al., 2023).

#### 5.2.2 SCFAs

SCFAs are mainly produced by the fermentation of undigested dietary fiber in the cecal and large intestinal by anaerobic microbiota and include acetate, butyrate, propionate, and so on (Macfarlane and Macfarlane, 2003). SCFAs are key regulators of lipid metabolism and inhibition of inflammatory responses (Brown and Hazen, 2018). SCFAs are produced by the metabolism of different GM. For example, *Bacteroides* produce acetate and propionate, while *Firmicute*s produce butyrate (Jia et al., 2023).

Current studies suggest that SCFAs have a potential modulatory effect on AS and are considered to be protective factors for AS. SCFAs are strongly associated with inhibition of intestinal inflammation, protection of intestinal barrier function, and improvement of lipid metabolism. Supplementation with a diet containing 1% butyrate reduced the size of aortic lesions by 50% in ApoE^−/−^ mice (Aguilar et al., 2014). SCFAs play a beneficial role in preventing vascular inflammation not only by activating G-protein coupled receptor 41/43 and inhibiting histone deacetylases to alleviate the endothelial inflammatory response (Li et al., 2018) but also by blocking the NLRP3/Caspase-1 pathway to inhibit the activation of macrophage (Yi et al., 2022). Propionate and butyrate alleviate the inflammatory response and exert immunomodulatory effects by inhibiting LPS-induced expression of TNF-α and nitric oxide synthase (NOS) by monocytes (Vinolo et al., 2011). Propionate also inhibits LPS or TNF-α-induced secretion of IL-6 and IL-8 from endothelial cells and reduces the expression of VCAM-1 (Li et al., 2018). In addition, in ApoE^−/−^ mice fed a high-fat diet (HFD), propionate suppressed the expression of Niemann-Pick C1-like 1 by increasing the number of regulatory T cells and IL-10 levels in the intestinal microenvironment and reduced intestinal cholesterol absorption, which in turn inhibited AS progression (Haghikia et al., 2022). A high-fiber diet alters GM by increasing the abundance of acetate-producing bacteria, which in turn protects against the development of cardiovascular disease (Marques et al., 2017). Transcriptome analysis showed that the protective effects of high fiber and acetate were associated with the downregulation of Egr1, a master regulator of cardiovascular inflammation (Marques et al., 2017). In mouse models of hypertensive cardiac injury and AS, propionate significantly reduced the lesion size in AS and attenuated systemic inflammation, as evidenced by reductions in splenic effector memory T-cell frequency and splenic T-helper 17 cells, as well as reductions in localized cardiac immune cell infiltration in mice in both models (Bartolomaeus et al., 2019). Moreover, due to their antioxidative properties, SCFAs can attenuate the oxidative and pro-inflammatory characteristics of AS. Butyrate attenuated endothelial dysfunction at AS lesion sites by inhibiting CCL-2 and superoxide ion productions and reducing the NADPH oxidase subunit p22phox (Aguilar et al., 2016). Co-culture with macrophages significantly reduced NOS, NO, and iNOS production, which effectively inhibited macrophage migration and activation (Aguilar et al., 2016). Butyrate also prevented endothelial dysfunction in AS by reducing endothelial NOX2 expression and ROS production via the PPARδ/miR-181b pathway and rescuing impaired endothelium-dependent relaxations in IL-1β-treated thoracic aorta ring (Tian et al., 2021). The above indicates that inhibition of oxidative stress may be one of the mechanisms of SCFAs-mediated anti-AS.

Additionally, modulation of lipid metabolism is another pathway of SCFAs against AS. Hypercholesterolemia is one of the risk factors for AS, and ABCA1 mediates the binding of cholesterol to apolipoprotein A-I (ApoA-I) to produce HDL (Li et al., 2020). This process is the main function of ABCA1 and a key step in HDL synthesis in the blood. Butyrate significantly inhibits HFD-induced AS and hepatic steatosis by a mechanism related to the promotion of ABCA1-mediated cholesterol efflux from macrophages (Du Y et al., 2020). SCFAs with two to four carbons lower cholesterol levels by up-regulating hepatic gene expression of sterol-regulatory-element-binding protein 2, low-density-lipoprotein receptor, and cholesterol 7α-hydroxylase to promote hepatic uptake of cholesterol from the blood and accelerate the excretion of bile acids in the feces (Zhao et al., 2017). The concentration of ApoA-I is reduced by inflammation, which may lead to HDL dysfunction and induce cardiovascular disease. SCFAs inhibit NF-κB activation through the peroxisome proliferator-activated receptor-alpha (PPARα) pathway, thereby rescuing the transcriptional function of ApoA-I under inflammatory conditions (Tayyeb et al., 2020). SCFAs have evolved as beneficial microbial products for the prevention or treatment of AS. The risk of AS can be reduced by increasing dietary fiber or direct SCFA supplementation, or the GM microenvironment can be improved by supplementing or transplanting SCFA-producing GM with increased SCFA-producing capacity.

### 5.3 Secondary bile acids

Bile acids are receiving increasing attention and focus from the medical community as important signaling factors produced by GM. Primary bile acids that escape the enterohepatic circulation are metabolized by GM into secondary bile acids (Sun et al., 2019). Secondary bile acids mainly include deoxycholic acid, litho biliary acid, and ursodeoxycholic acid. In mice, secondary bile acids have been shown to regulate lipid metabolism by modulating specific genes in the liver or small intestine (Mills et al., 2019). A study have found that under the same dietary conditions, the bile acid excretion ability of patients with a high-cholesterol diet is significantly weaker than that of healthy controls, the content of total bile acids in feces is significantly less than that of controls, and DCA and LCA are less (Charach et al., 2018), which suggest that the inability to excrete bile acids effectively accelerates the development of AS.

Secondary bile acids act as important signaling factors that regulate lipid, glucose, and energy metabolism, inflammatory responses, and immune responses mainly through the regulation of FXR and G-protein-coupled bile acid receptor 5 (TGR5) (Kawamata et al., 2003; Chavez-Talavera et al., 2017). Activation of FXR promotes RCT, which has been shown to increase fecal cholesterol excretion and downregulate proinflammatory cytokine levels (Hageman et al., 2010). After feeding on a high-fat diet, the area of AS lesions in mice deficient in both FXR and ApoE-deficient mice was approximately twice as large as in ApoE-deficient mice only (Hanniman et al., 2005). This suggests that the expression of FXR has a protective role in AS. In addition, FXR also inhibits the activation of NF-κB and the expression of inducible NOS, thereby inhibiting vascular smooth muscle migration, preventing vascular remodeling, and enhancing the stability of AS plaques (Li et al., 2007). However, another study found that FXR deficiency reduced the area of AS lesions in male Ldlr^−/−^ mice and hypothesized that the mechanism was related to the inhibition of CD36-dependent lipid uptake in macrophages (Zhang et al., 2006). Notably, the expression of FOM3 is regulated by FXR, indicating that the metabolic pathways of TMAO and bile acids may interact (Bennett et al., 2013). TGR5 is an important receptor for bile acid metabolism (Chiang and Ferrell, 2019). Bile acids reduce the transcriptional activity of NF-κB by activating the cell surface membrane receptor TGR5 to downregulate the levels of pro-inflammatory cytokines, which in turn inhibits inflammation in AS plaques of Ldlr^−/−^ mice and reduces the content of macrophages and lipid load in the plaques (Pols et al., 2011). In addition, TGR5 agonism induces NO production via Akt activation and intracellular Ca^2+^ increase, and this function inhibits the adhesion of monocytes to endothelial cells in response to inflammatory stimuli (Kida et al., 2013).

## 6 Natural products modulate the crosstalk between GM and immune response in AS

Natural products have been the subject of many studies and they are increasingly being used as a complementary therapy in clinical practice. It is well known that most natural products are chemically diverse and bioavailable. To maximize the therapeutic potential of natural products, a large number of trials are still in progress or preparation. Natural products have been shown to have multi-pathway and multi-target therapeutic effects in the treatment of AS (Liu et al., 2022). In recent years, some scholars have turned their attention to the study of natural products and GM and tried to acknowledge the pharmacological activities of natural products from the perspective of GM. The role of GM in AS has been gradually unveiled (Verhaar et al., 2020). So, is the specific mechanism of natural products in the treatment of AS by targeting GM and immune response crosstalk? Recent studies have provided an answer. As shown in Table 1.

**TABLE 1:** Mechanism of natural products targeting the GM-immune response axis in the treatment of AS.

Active metabolites	Natural sources	Experimental model	Regulation of GM and its metabolism	Targeting the immune inflammatory response	Reference
Resveratrol	*Reynoutria japonica* Houtt [Polygonaceae; *Polygonum cuspidatum Sieb et zucc*]	ApoE^−/−^ mice, RAW264.7 cells	• Reduction of intestinal fatty acids and monoglycerides accumulation	• Activation of the PPARα/γ signaling pathway accelerates ABCA1/G1-mediated cholesterol efflux and inhibits the accumulation of total cholesterol, esterified cholesterol, and neutral lipids in macrophages	Ye et al. (2019)
		ApoE^−/−^ mice	• Increasing the relative abundance of *Bacteroides, Lactobacillus, Bifidobacterium*, and *Akkermansia*	-	Chen et al. (2016)
			• Decreasing the relative abundance of *Prevotella, Ruminococcaceae, Anaerotruncus, Alistipes, Helicobacter*, and *Peptococcaceae*		
			• Enhancement of bile acid synthesis in the liver via the enterohepatic FXR-FGF15 axis		
			• Inhibition of TMAO production		
Berberine	*Coptis chinensis* Franch [Ranunculaceae; *Coptidis rhizoma*]	ApoE^−/−^ mice	• Increasing the relative abundance of *Roseburia, Blautia, Allobaculum, Alistipes*, and *Turicibacter*	• Decreased the levels of TNF-α, IL-1β, and IL-6 and significantly increased the level of IL-10 to inhibit the inflammatory response	Wu et al. (2020)
			• Upregulation of SCFAs production and inhibition of TMAO production		
Berberine	*Phellodendron amurense* Rupr [Rutaceae; *Phellodendri chinensis cortex*]	SD rats and hamsters	• Increasing the relative abundance of *Allobaculum*, *Akkermansia,* and *Lachnospiraceae*	-	Ma et al. (2022)
			• Decreasing the relative abundance of *Eubacterium, Ruminococcaceae, Prevotellaceae, Ruminoccoccus*, and *Jeotgalicoccus*		
			• Reduction of TMAO production		
		ApoE^−/−^ mice	• Increasing the relative abundance of *Lachnospiraceae, Bacteroidales*, and *Eubacterium*	-	Li et al. (2021)
			• Reduction of TMAO production		
Curcumin	*Curcuma longa* L [Zingiberaceae; *Curcumae longae* ruizoma]	ApoE^−/−^ mice	• Recovery of *Bacteroidetes/Firmicutes* ratio	• Promotion of M2 macrophage polarization and inhibition of M1 macrophage polarization	Zhang et al. (2022)
			• Increasing the relative abundance of *Verrucomicrobia* and *Akkermansi*	• Downregulation of NF-κB p65 and NLRP3 expression	
			• Decreasing the relative abundance of *Lactobacillus*	• Downregulation of IL-1β and IL-6 levels	
			• Reduction of TMAO production		
Curcumin		LDLR^−/−^ mice	• Suppression of LPS production	• Inhibiting the activation of macrophages and reducing the release of IL-6 and MCP-1 from macrophages	Ghosh et al. (2014)
				• Downregulation of NF-κB p65 expression	
Quercetin	*Scutellaria baicalensis* Georgi [Lamiaceae; *Scutellariae radix*]	ApoE^−/−^ mice	• Decreasing the relative abundance of *Phascolarctobacterium* and *Anaerovibrio*	• Downregulation of TNF-α and IL-6 levels	Wu et al. (2019)
			• Promoting the synthesis of primary bile acids		
		Ldlr^−/−^ mice	• Increasing the relative abundance of *Actinobacteria* and *Bacteroidetes*, decreasing the relative abundance of *Firmicutes*	• Downregulation of IL-6 levels	Nie et al. (2019)
			• Increasing the relative abundance of A*kkermansia, Bacteroides, Parabacteroides* and *Ruminococcus*		
			• Decreasing the relative abundance of *Lactobacillus*		
			• Increasing the metabolism of fecal sterols		
Naringin	*Drynaria roosii* Nakaike [Polypodiaceae; *Drynariae rhizome*]	ApoE^−/−^ mice	• Increasing the relative abundance of *Firmicutes*		Wang et al. (2020)
			• Decreasing the relative abundance of *Bacteroidetes* and *Verrucomirobia*	• Inhibition of macrophage infiltration and reduction of the formation of foam cell	
			• Increasing the relative abundance of *Eubacterium, decreasing the relative abundance of Bacteroides, Bifidobacterium, Lactococcus, Clostridium sensu stricto, Streptococcus, Desulfovibrio, Parasutterell*a, and *Bacteroides*	• Downregulation of TNF-α levels	
			• Increasing the production of secondary bile acids		
Ginsenoside Rc	*Panax ginseng* C.A.Mey [Araliaceae; *Ginseng radix et rhizome*]	ApoE^−/−^ mice	• Increasing the relative abundance of *Bacteroidetes*	• Downregulation of TNF-α, IL-1β and IL-6 levels	Xie et al. (2022)
			• Decreasing the relative abundance of *Firmicutes*		
			• Increasing the relative abundance of *Muribaculaceae, Ileibacterium, Lactobacillus* and *Bifidobacterium*		
			• Decreasing the relative abundance of *Faecalibaculum, Eubacterium, Oscillibacter* and *Blautia*		
			• Increasing the production of primary bile acids		
Paeonol	*Paeonia* × *suffruticosa*	ApoE^−/−^ mice	• Increasing the production of SCFAs	• Reducing the number of CD4^+^IL17A^+^ (Th17) and increasing the number of CD4^+^CD25^+^Foxp3+ (Treg)	Shi et al. (2021)
	Andrews [Paeoniaceae; *Moutan cortex*]			• Regulating the balance of Treg/Th17	
	*Vincetoxicum mukdenense* Kitag [Apocynaceae; *Cynanchi paniculate radix et rhizoma*]			• Downregulation of IL-1β, IL-6, TNF-α, and IL-17A levels, upregulation of IL-10 levels	
Millet shell polyphenols	*Fagopyrum esculentum* Moench [Polygonaceae; *Fagopyrum esculentum* Moench]	ApoE^−/−^ mice	• Increasing the relative abundance of *Bacteroidetes*	• Downregulation of TNF-α and IL-1β levels	Liu et al. (2021)
			• Decreasing the relative abundance of *Verrucomicrobia* and *Actinobacteria*		
			• Increasing the relative abundance of *Oscillospira* and *Ruminococcus*		
			• Ddecreasing the relative abundance of *Allobaculum*		
Flaxseed oil	*Linum usitatissimum* L [Linaceae; *Lini semen*]	ApoE^−/−^ mice	• Reduction of the ratio of *Firmicutes*/*Bacteroidetes*	• Downregulation of the level of F4/80^+^CD11b^+^ cells	Li et al. (2022)
			• Decreasing the relative abundance of *Intestinimonas, Bilophila, Anaerotruncus, Oscillibacter, Negativibacillus, Lachnoclostridium*, and *Enterorhabdus*	• Downregulation of TNF-α, IL-1β and IL-6 levels	
			• Increasing the production of SCFAs	• Downregulation of MCP-1 level	
Capsaicin	*Capsicum annuum* L [Solanaceae; *Capsici fructus*]	ApoE^−/−^ mice	• Increasing the relative abundance of *Deferribacteres*	• Downregulation of IL-6 level	Dai et al. (2022)
			• Decreasing the relative abundance of *Cyanobacteria* and *Tenericutes*		
			• Increasing the relative abundance of *Ileibacterium, Ruminococcacee, Odoribacter,* and *Mucispirillum*		
			• Decreasing the relative abundance of *Faecalibaculum* and *Marvinbryantia*		
Gastrodia extract	*Gastrodia elata* Blume [Orchidaceae; *Gastrodiae rhizome*]	C57BL/6J mice	• Increasing the relative abundance of *Bacteroidetes* and *Actinobacteria*	• Downregulation of ICAM-1, IL-1β and IL-8 levels	Liu et al. (2021)
			• Decreasing the relative abundance of *Proteobacteria* and *Firmicutes*	• Upregulation of IL-10 levels	
			• Increasing the production of SCFAs	• Upregulation of IgA levels	
Gastrodin	*Gastrodia elata* Blume [Orchidaceae; *Gastrodiae rhizome*]	C57BL/6J mice	• Increasing the relative abundance of *Bacteroidetes, Actinobacteria,* and *Firmicute*	• Downregulation of TNF-α, ICAM-1, IL-1β, IL-8 and IL-6 levels	Liu et al. (2021)
			• ;Decreasing the relative abundance of *Proteobacteria*	• Upregulation of IL-10 levels	
			• Increasing the production of SCFAs	• Upregulation of IgA levels	
Gypenoside XLIX	*Gynostemma pentaphyllum* (Thunb.) Makino [Cucurbitaceae; *Gynostemma pentaphyllum* (Thunb.) Makino]	ApoE^−/−^ mice	• Increasing the ratio of *Bacteroidetes/Firmicutes*	• Downregulation of IL-6, IL-1β, TNF-α, ICAM-1 and CCl2 expression	Gao et al. (2022)
			• Increasing the relative abundance of *Bacteroidetes* and *Firmicutes*		
			• Increasing the relative abundance of *Eubacterium*, *Roseburia*, *Bifidobacterium*, *Lactobacillus*, and *Prevotella*		
			• Decreasing the relative abundance of *Clostridioides* and *Desulfovibrionaceae*		
			·Increasing the production of SCFAs		
			·Decreasing the production of TMA/TMAO		

### 6.1 Resveratrol

Resveratrol (RSV) is an antitoxin produced by plants in nature when they are stimulated. It belongs to a polyphenolic organic metabolite and is widely found in plants such as *Polygonum cuspidatum* (Meng et al., 2023). Resveratrol has various pharmacological effects such as anti-inflammatory, antibacterial, antioxidant, and tumor-inhibiting activities (Koushki et al., 2018). The protective effect of resveratrol in AS-related diseases has been demonstrated (Koushki et al., 2018). RSV increases the levels of *Lactobacillus* and *Bifidobacterium* in the intestine of C57BL/6J and ApoE^−/−^ mice, thereby enhancing the activity of bile saline lyase and promoting the unbinding of bile acids and fecal excretion (Chen et al., 2016). In addition, RSV reduces the GM metabolism of TMAO and enhances bile acid synthesis in the liver via the enterohepatic FXR-FGF15 axis (Chen et al., 2016). The intestinal carbohydrate and amino acid metabolism were effectively improved and the accumulation of fatty acids and monoglycerides was significantly reduced in AS mice successfully modeled by a high-fat diet and fed RSV (Ye et al., 2019). Peroxisome proliferator-activated receptors (PPARs) play a key role in cholesterol homeostasis and are an important way to treat dyslipidemia in AS (Hu et al., 2022). RSV promotes ABCA1/G1-mediated cholesterol efflux through activation of the PPARα/γ signaling pathway and inhibits the oleic acid-induced accumulation of total cholesterol, esterified cholesterol, and neutral lipids in macrophages (Ye et al., 2019). These results suggest that RSV interacts with GM and thus plays a regulatory role in AS.

### 6.2 Berberine

Berberine (BBR) is a traditional Chinese medicinal preparation containing isoquinoline alkaloids extracted from plants, which has a variety of pharmacological properties (Wang et al., 2019). In China, it has been used to treat diabetes, obesity, AS, and metabolic diseases (Zhang et al., 2020). BBR was found to inhibit *Streptococcus*, *Staphylococcus epidermidis,* and *Shigella dysenteriae* (Wang et al., 2009; Yan et al., 2014), and increase the abundance of *Lactobacillus* and *mucinophilic Ackermannia* in the intestine of mice (Zhang et al., 2019). Therefore, BBR is considered to have antibiotic-like effects and a broad antibacterial spectrum.

The metabolite of BBR, dihydro flavopiridol, inhibits TMAO production by blocking the Choline-TMA-TMAO production pathway through vitamin-like effects, leading to a decrease in serum TMAO levels and contributing to the inhibition of plaque formation in blood vessels (Ma et al., 2022). Clinical trials have confirmed that both TMA and TMAO in the feces of patients were significantly reduced after 4 months of oral BBR, and plaque scores were significantly decreased (Ma et al., 2022). The mechanism is related to the reduction of cutC and cntA, key functional genes of the TMA synthesis pathway by BBR, as a way to reshape the GM and thus inhibit the development of AS (Li et al., 2021). BBR showed a dose-dependent approach in reducing plaque area and improving the inflammatory response (Wang et al., 2009). Compared to low-dose (50 mg/kg) BBR, high-dose (100 mg/kg) BBR significantly reduced plaque size, significantly reduced the levels of TNF-α, IL-1β, and IL-6, and significantly increased the levels of IL-10. However, low-dose BBR had no significant effect on IL-6 and IL-10 levels. In addition, alterations in the composition and function of the GM showed different sensitivities to BBR doses. For example, *Alistipes* and *Roseburia* were significantly enriched in the high-dose BBR group, while *Blautia* and *Allobaculum* were more enriched in the low-dose group.

### 6.3 Curcumin

Curcumin has multiple pharmacological mechanisms such as anti-inflammatory, antioxidant, and anti-free radical (Peng et al., 2021). Numerous studies have shown that curcumin can play a positive role in the prevention and treatment of many metabolic diseases by interacting with GM to achieve beneficial effects on the health of the host organism (Pluta et al., 2020). The study showed found that after 15 days of continuous gavage of C57BL/6 mice with curcumin (100 mg/kg), the composition of the GM of mice was significantly altered, with a significant decrease in the abundance of pathogenic bacteria such as *Prevoteltellaceae*, *Bacteroidaceae* and *Rikenellaceae* (Shen et al., 2017). An 8-week randomized, double-blind, and placebo-controlled trial (Peterson et al., 2018) showed an overall decrease of 15% in bacterial species in the placebo group after treatment, compared to an increase of 7% and 69% in the turmeric and curcumin-treated groups, respectively. The above suggests that curcumin has a significant modulating effect on the composition of GM.

Curcumin has been confirmed to directly affect GM and restore cadmium-induced metabolic disorders of GM, such as restoring the *Bacteroidetes*-to-*Firmicutes* ratio, reducing the abundance of *Lactobacillus* and increasing the abundance of *Verrucomicrobia* (Zhang et al., 2022). Curcumin restored the polarization of macrophages by remodeling the GM, that is, promoting M2-type polarization and inhibiting M1-type polarization (Zhang et al., 2022). In addition, in the AS region of the aorta, curcumin downregulated the expression of the inflammatory proteins NF-κB p65 and NLRP3 and decreased the levels of the inflammatory factors IL-1β and IL-6 (Zhang et al., 2022). The pathological increase of LPS can promote the hyperactivation of macrophages and activate the expression of NF-κB, upregulate the levels of chemokines and proinflammatory cytokines, which in turn lead to inflammation-associated diseases (Sieve et al., 2018). The disruption of the intestinal barrier loses the ability to limit the release of LPS from bacteria into circulation (Wang et al., 2017). Curcumin inhibited LPS-induced translocation of [^3^H]-mannitol and FITC dextran by increasing the activity of intestinal mucosal defense factor intestinal alkaline phosphatase (IAP), which in turn improved intestinal barrier function (Ghosh et al., 2014). The mechanism by which curcumin inhibits AS may be through reducing the release of LPS and inhibiting the release of proinflammatory factors such as IL-6 and MCP-1 after excessive activation of macrophages, and reducing the expression of pro-inflammatory transcription factor NF-κB.

Curcumin not only achieves its broad biological functions by regulating the level of structural abundance of the host GM but also is metabolized and catabolized by the microbiota in the host gut to produce a variety of active metabolites that participate in the regulation of various physiological processes in the host (Tsuda, 2018). The human GM catabolizes curcumin mainly through several pathways such as reduction, demethylation, acetylation, and hydroxylation, producing a mixture of demethoxycurcumin and bisdemethoxycurcumin (Lou et al., 2015). Among them, bisdemethoxycurcumin can significantly increase the relative abundance of *E. faecalis* and decrease the relative abundance of *Bacteroides* and *Subdoligranulum* in the broiler intestine, as well as significantly improve the morphology of the intestinal mucosa, maintain the integrity of the intestinal barrier, inhibit the secretion of pro-inflammatory factors, and promote the recovery of GM dysbiosis, thereby alleviating LPS-induced intestinal inflammatory damage in broilers (Zhang et al., 2021). This suggests that the interaction between curcumin and GM is an important link in regulating the physiological functions of the organism.

### 6.4 Quercetin

Numerous *in vivo* and *in vitro* studies have shown that quercetin exhibits good protective effects on several key aspects of AS development, such as reducing oxidative stress levels, antagonizing inflammation, and restoring vascular endothelial function (Xiao et al., 2017; Patel et al., 2018). Pharmacokinetic studies show that the bioavailability of oral quercetin is only 5.3%, of which about 93.3% is metabolized in the gut (Chen et al., 2005).

Quercetin can selectively promote the proliferation of beneficial intestinal bacteria and inhibit the colonization of harmful bacteria in the gut, thereby optimizing the intestinal microecological environment to maintain the health of the gut and the body. Quercetin significantly increased the alpha diversity of GM. At the phylum level, quercetin increased the relative abundance of *Actinobacteria*, *Cyanobacteria*, and *Firmicutes* and decreased the relative abundance of *Verrocomicrobia* (Nie et al., 2019; Wu et al., 2019). At the genus level, the abundance of *Ackermannia*, *Bacillus*, *Paramecium*, and *Ruminalis* was significantly increased by quercetin treatment, while the abundance of *Lactobacillus* was significantly decreased (Nie et al., 2019).

Quercetin not only regulates the abundance of GM but also influences the production of metabolites of GM. Quercetin promotes the synthesis of primary bile acids, which in turn consumes more cholesterol and reduces the clinical risk of HFD-induced AS (Wu et al., 2019). Furthermore, reducing the abundance of *Actinobacteria* and increasing the abundance of *Firmicutes* increased the level of IL-6 and inhibited the expression of malondialdehyde (MDA), which in turn had a positive regulatory effect on oxidative stress, immunity, and inflammatory response (Nie et al., 2019). The above studies suggest that quercetin plays a beneficial role in GM and immune response crosstalk, but the specific mechanisms still need to be further studied.

### 6.5 Naringin

Naringin is a natural dihydro flavonoid mainly derived from the peel and pulp of Rutaceae. Naringin has been reported to inhibit AS by inhibiting the adhesion of monocytes and endothelial cells (Hsueh et al., 2016), inhibiting macrophage infiltration, and regulating cholesterol metabolism in macrophages (Xu et al., 2019). Naringin has low bioavailability and is difficult to digest and absorb in the small intestine, thus reaching the colon where the GM colonizes (Mulvihill et al., 2016). Therefore, oral administration of naringin may affect the composition and metabolism of GM. Naringin significantly reduced the serum levels of total cholesterol, low-density lipoprotein cholesterol, very-low-density lipoprotein cholesterol, and free cholesterol in AS model mice and reduced the area of AS plaques (Wang et al., 2020). Meanwhile, naringin also promoted the diversity and stability of GM, reduced the number of harmful microbiota, and increased the number of beneficial microbiota. For example, increasing the abundance of *Firmicutes* and reducing the abundance of *Bacteroidetes* and *Verrucomirobia*, thus promoting the synthesis of secondary bile acids. In terms of lipid metabolism, naringin regulated cholesterol metabolism by inhibiting the liver X receptor and the steroid transporter protein ABCG5/G8. In addition, naringin inhibited macrophage infiltration in AS plaques, which in turn alleviated the inflammatory response.

### 6.6 Ginsenoside Rc

Ginsenoside Rc is one of the bioactive components in ginseng with a wide range of physiological activities, such as inhibition of oxidative stress, anti-inflammation, and regulation of glucose metabolism (Lee et al., 2010; Kim et al., 2014; Yu et al., 2017). Ginsenoside Rc also showed anti-atherosclerosis activity. Ginsenoside Rc significantly influenced the composition of GM and fecal metabolites in mice with high-fat diet-induced AS, thereby improving the condition of AS. Specifically, ginsenoside Rc increased the number of beneficial bacteria such as *Lactobacillus* and *Bifidobacterium*, while decreasing the number of harmful microbiota such as *Faecalibaculum* (Xie et al., 2022). In addition, ginsenoside Rc increases the metabolism of primary bile acids by remodeling the GM, which in turn inhibits the expression of the pro-inflammatory cytokines TNF-α, IL-6, and IL-1β to alleviate inflammatory response (Xie et al., 2022).

### 6.7 Paeonol

Paeonol is a bioactive substance extracted from the root bark of a *Paeonia ×suffruticosa* Andrews in Ranunculaceae or the dried root of *Cynanchum paniculatum* in Metaplexy. It has analgesic, anti-inflammatory, and anti-tumor pharmacological effects (Zhang et al., 2019). Current studies found that Paeonol inhibits the progression of AS by downregulating the inflammatory response of vascular endothelial cells (Yu et al., 2020), inhibiting excessive proliferation of VSMCs (Zhang et al., 2018), and regulating macrophage function (Xu et al., 2018). Paeonol can alleviate AS in a GM-dependent manner. Paeonol inhibits plaque formation and vascular fibrosis in a high-fat diet-induced AS mouse model (Shi et al., 2021). Paeonol selectively upregulates the proportion of Treg cells and reduces the number of Th17 by remodeling the GM, such as increasing the metabolism of SCFAs and reducing the accumulation of LPS (Shi et al., 2021), and inhibits the abnormal proliferation of VSMCs caused by the high expression of osteopontin in monocytes (Shi et al., 2022). In addition, Paeonol significantly inhibited the expression of inflammatory cytokines IL-1β, IL-6, TNF-α, and IL-17A and increased the level of anti-inflammatory cytokine IL10 in the aorta of AS mice (Shi et al., 2022).

### 6.8 Millet shell polyphenols

As an important component of cereals, the active polyphenols in millet shells have hypolipidemic and antioxidant effects as well as inhibiting inflammatory responses (Khare et al., 2020). These functions are closely related to their anti-AS effects. Millet shell polyphenols inhibited aortic lesions in ApoE^−/−^ mice (Liu et al., 2021). Its mechanism is to regulate the relative abundance of GM, such as reducing the abundance of *Verrurella* and *Actinomyces* and increasing the abundance of *Bacteroidetes*, which in turn exerts a barrier-protective effect and inhibits the expression of LPS and the pro-inflammatory cytokines TNF-α and IL-1β (Liu et al., 2021). In addition, Millet shell polyphenols block the expression of STAT1 and NF-κB in macrophages and inhibit the formation of macrophage-derived foam cells (Liu et al., 2023).

### 6.9 Flaxseed oil

Flaxseed oil, one of the major edible oils worldwide, is the main component extracted from *Linum usitatissimum* L and an important source of α-linolenic acid (Xu et al., 2012). As an important nutrient for humans, flaxseed oil is closely related to vision, brain development, and behavioral development. In addition, flaxseed oil plays an active role in lowering blood lipids and blood glucose, antioxidant, antibacterial and anti-inflammatory, anti-cancer, and anti-AS (Dupasquier et al., 2007; Zhu et al., 2020; Bao et al., 2022). Flaxseed oil reduced the relative abundance of *Enterobacteriacea*e, *Biliophilus*, *Anaerobacter*, *Oscillibacter*, and *Negatibacillus* in the GM of AS model mice and significantly reduced the proportion of the *Firmicutes*/*Bacteroidetes* ratio (Li et al., 2022). The production of SCFAs, a metabolite of GM in mice, was also promoted by flaxseed oil (Li et al., 2022).

By modulating the GM of AS model mice, flaxseed oil downregulated the levels of F4/80^+^CD11b^+^ cells in the aortic region and suppressed the inflammatory response (such as TNF-α and IL-1β), thus exerting an anti-AS effect (Li et al., 2022).

### 6.10 Capsaicin

Capsaicin is a vanilla amide-containing alkaloid and the natural capsaicin consists of a series of similar substances such as dihydrocapsaicin, hypo-dihydrocapsaicin, and hypercapsaicin (Wang et al., 2022). Among them, capsaicin which can be absorbed and metabolized by the gastrointestinal tract accounts for 80%–90%. In recent years, studies have shown that capsaicin has a wide range of pharmacological effects, including analgesia, regulation of blood glucose metabolism, and antioxidant and anti-AS effects (Munjuluri et al., 2021; Wang et al., 2022). It was found that capsaicin exerts anti-AS effects by modulating the GM of ApoE^−/−^ mice as well as metabolites such as increasing the abundance of *Turicibacter*, *Odoribacter*, and *Ileibacterium* in the feces and reducing the metabolism of deoxycholic acid, bile acids, hypoxanthine, and fecal bile in the cecum, thereby inhibiting the inflammatory response (Dai et al., 2022).

### 6.11 Gastrodia extract and gastrodin

*Gastrodia elata* is a traditional Chinese medicine with a long history of application in China. Studies have shown that *Gastrodia elata* has good efficacy in the treatment of cardiovascular diseases (Sun et al., 2023). Gastrodin is the most important active component of *Gastrodia elata*. Gastrodin and gastrodin extracts reduce lipid deposition in early AS mice in a dose-dependent manner and slow down the progression of AS (Liu et al., 2021b; Liu et al., 2021). The mechanism is to increase the diversity of GM, regulate the relative abundance of GM (such as increasing the relative abundance of *Bacteroidetes* and reducing the relative abundance of *Proteobacteria*), and increase the production of SCFAs, thereby inhibiting the secretion of the pro-inflammatory cytokines IL-1β and IL-6 and promoting the expression of the anti-inflammatory cytokine IL-10, and ultimately alleviating the inflammatory response in AS mice (Liu et al., 2021b; Liu et al., 2021). Asparagine and asparagine extracts also increase sIgA levels and downregulate D-LA expression, which in turn promote the repair of GM and reduce permeability (Liu et al., 2021). This also helps to delay the progression of AS. In addition, gastrodin reduces the formation of foam cells and improves inflammation by inhibiting the NF-κB signaling pathway to anti-AS (Xue et al., 2023).

### 6.12 Gypenoside XLIX

*Gynostemma pentaphyllum* (Thunb.) Makino is a perennial prostrate plant in the Cucurbitaceae family, which is mainly distributed in China, Korea, and Japan. *Gynostemma pentaphyllum* (Thunb.) Makino has been used as a Chinese herbal medicine in ancient times, which has the functions of clearing heat and detoxifying, invigorating the spleen and calming the mind, invigorating qi, and promoting body fluid (Nguyen et al., 2021). With the discovery of modern research on herbal medicine, *Gynostemma pentaphyllum* (Thunb.) Makino leaves have been widely used as vegetables, dietary supplements, and herbal teas for their potential in reducing hyperlipidemia, malignancy, diabetes, chronic inflammation, and anti-aging (Liao et al., 2021; Weng et al., 2021). As the main chemical component of *Gynostemma pentaphyllum* (Thunb.) Makino, Gypenoside XLIX inhibits the inflammatory response in AS mice by modulating the GM (such as increasing the relative abundance of *Firmicutes*, *Bacteroidetes*, *Bifidobacteria*, and decreasing the relative abundance of *Clostridioides* and *Desulfovibrionaceae*), inhibiting the production of TMAO and promoting the synthesis of SCFAs (Gao et al., 2022).

### 7 Oral probiotics modulate the crosstalk between GM and immune responses in AS

Probiotics colonized in the gut, such as *Lactic Acid Bacteria* and *Bifidobacterium*, can positively affect human health by improving GM balance, enhancing immune function, and regulating metabolism (Hill et al., 2014). GM imbalance can lead to intestinal environment disorder, resulting in the reduction of the total amount of probiotics and causing a series of diseases. Current studies have shown that probiotic supplementation can effectively improve the risk factors of AS, such as lipid metabolism disorders, hypertension, and persistent inflammation. The intervention of probiotics provides a new direction and idea for the regulation and remodeling of intestinal microorganisms. Oral probiotics have opened new avenues for the treatment of AS.

The interactions between probiotics and the host immune system are highly complex and are still being explored. Treatment of ApoE^−/−^ mice with the probiotic *lactic acid bacterium Pediococcus acidilactici* R037 found that the number of CD4^+^T cells producing interferon-γ in the spleen and the production of proinflammatory cytokines by splenic lymphocytes were significantly reduced (Mizoguchi et al., 2017). *In vitro*, experiments further confirmed that R037 alleviated AS by inducing tolerogenic DCs, thereby inhibiting Th1-driven inflammation and the proliferation of CD4^+^T cells (Mizoguchi et al., 2017). Lab4 is a probiotic consortium composed of *Lactobacillus acidophilus* CUL21 (NCIMB 30156) and CUL60 (NCIMB 30157), *Bifidobacterium bifidum* CUL20 (NCIMB 30153) and *Bifidobacterium animalis subsp. lactis* CUL34 (NCIMB 30172). The combination of Lab4 with *L. plantarum* CUL66 (called Lab4P) significantly reduced LDL and very low-density lipoprotein levels in HFD-fed LDL receptor-deficient mice. It alleviates inflammation by inhibiting monocyte migration, the proliferation of monocytes and macrophages, and reduces the formation of foam cells (O'Morain et al., 2021). In addition, Lab4P can increase the number of VSMCs, which is a marker of plaque stability (O'Morain et al., 2021). VSL#3 is a well-studied probiotic mixture comprised of 8 strains, showing similar efficacy to telmisartan in inhibiting vascular inflammation and reducing markers of AS development, and increasing the diversity of GM (Chan et al., 2016). A recent clinical study showed that supplementation with *Lactobacillus paracasei* TISTR 2593 significantly reduced MDA and TNF-α levels in AS patients, thereby inhibiting oxidative stress and inflammation (Khongrum et al., 2023). It also significantly increased serum ApoE and adiponectin levels (Khongrum et al., 2023). It should be noted that not all probiotics have anti-AS effects, such AS *Lactobacillus reuteri*, which does no effect on AS in ApoE^−/−^ mice (Fak and Backhed, 2012).

In addition, probiotics have been shown to exert positive effects by influencing the production of gut microbiota metabolites. *Bifidobacterium animalis subsp. lactis* F1-three to two, *Bifidobacterium animalis subsp. lactis* F1-7, *Lactobacillus plantarum* F3-2 reduce the content of TMA in the intestine and downregulate the levels of TMA and TMAO in serum, and the mechanism is not related to the regulation of FMO3 function (Liang et al., 2020). The mechanism of action of this probiotic may be directly degrading TMA or changing the structure of GM. Similarly, *Bifidobacterium breve* Bb4 and *Bifidobacterium longum* BL1 and BL7 also reduced plasma TMAO and plasma and cecal TMA concentrations. However, it did not affect the protein expression of FMO, FMO3, and FXR and the excretion of TMAO (Wang et al., 2022). A Pilot Study found that short-term supplementation with probiotics *Streptococcus thermophilus* (KB19), *Lactobacillus acidophilus* (KB27), and *Bifidobacteria longum* (KB31) did not affect plasma TMAO levels in hemodialysis patients (Borges et al., 2019). Food-derived probiotics can increase the number of SCFAs-producing bacteria and promote the synthesis of SCFAs, which can delay the progression of AS (Derrien and van Hylckama, 2015). *Lactobacillus fermentum* ZJUIDS06 supplementation upregulated colon SCFA levels and increased the relative abundance of *Paracbacteroides* in the cecum of hyperlipidemic hamsters. This is a biomarker of colonic SCFA production and improved serum cholesterol levels (Yang et al., 2021). *Lactobacillus reuteri* CCFM8631 increased the contents of acetate, propionate, and butyrate and decreased the level of TMAO in the feces of AS model mice. At the same time, it improved the GM microenvironment (an increase in the relative abundance of faecal *Deferribacteres*, *Lachnospiraceae* NK4A136 group, *Lactobacillus,* and *Dubosiella*; a decrease in the relative abundance of *Erysipelatoclostridium* and *Romboutsia*) (Wang et al., 2022). The application of probiotics to improve health or treat diseases by affecting the physiological functions of the host has become a popular research area in medical disciplines. The current research has made preliminary progress, but limited by the current technology and the lack of prior knowledge, scientists have not yet fully revealed the interaction mechanism between each strain and between the microbiota and the host, which is the focus of GM research.

### 8 Dietary fiber in the prevention of AS

Dietary fiber is a non-digestible and non-absorbable carbohydrate polymer that can regulate the physiological health of the host by regulating the GM, promoting the dynamic balance of the intestinal microbiota, and then influencing the production of intestinal microbial metabolites. Consuming an abundance of fibrous foods, such as vegetables, fruits, grains, and legumes, is beneficial to gut health and overall health, and is thought to prevent cardiovascular disease. A cross-sectional study in Korea showed that total fiber and fruit fiber intake in men were inversely associated with metabolic syndrome, and increased total fruit fiber intake in women contributed to lower rates of obesity and abdominal obesity (Song and Song, 2021). Clinical studies have found that dietary fiber consumption can help regulate lipid metabolism and slow the progression of AS (Kameyama et al., 2020; Munoz-Cabrejas et al., 2021).

The fermentation of dietary fiber by GM produces large amounts of SCFAs, and the amount and type of SCFAs produced may vary due to different fermentation characteristics. For example, pea fiber significantly increases butyrate levels in humans (Guo et al., 2021). Whole wheat diets, on the other hand, have been associated with an increase in propionate (Vetrani et al., 2016). Dietary fiber may exert beneficial effects against AS by increasing the production of SCFAs. Soluble dietary fiber was found to remodel the microenvironment of GM by increasing beneficial intestinal bacteria and inhibiting TMAO production by activating AMPK (Li et al., 2017). In addition, the intake of dietary fiber helps to expand the size of the bile acid pool. Cereal fiber intake significantly increased serum bile acid concentrations in obese but not overweight individuals, which may be related to a compensatory increase in the bile acid pool (Weickert et al., 2018). Arabinoxylan and β-glucan significantly increased the number of bacilli in the intestinal tract of mice and significantly upregulated various genes involved in cholesterol and bile acid metabolism in the liver, which effectively promoted bile acid excretion and attenuated hepatic steatosis (Raza et al., 2019). Numerous current studies have confirmed the need for appropriate dietary modifications. Different dietary fibers have different regulatory effects on GM and their metabolites, requiring individualized programs for different populations and different diets.

## 9 Discussion

At present, although the mechanisms underlying the development of AS have not been elucidated, there has been some progress in related studies. The important role of immune response in AS has been widely recognized (Wolf and Ley, 2019). The immune response has a very complex biological characteristic, involving a variety of cell types, multiple pathway regulation, and diverse response types. The occurrence of immune responses depends on the recognition and activation of immune cells and the immune response. During the development and progression of AS, a complex series of changes in the local microenvironment occurs with the infiltration of multiple immune cells (Herrero-Fernandez et al., 2019). So, who can play a role in activating the immune response in AS? Numerous studies have found that GM and its metabolites are closely associated with AS (El et al., 2023). The GM constitutes a complex and vast microenvironment in the human body, and the composition and function of the microbiota affect the balance of health and disease.

Current research has shown that intervention with GM is an effective strategy for the prevention and treatment of AS. Firstly, targeting the GM itself, various compounds including natural product supplements have the potential to cause GM remodeling due to the sensitivity of the microbiota to changes in the chemical environment in the gut. Therefore, targeting GM remodeling is a way to explore the therapeutic potential of GM. As mentioned above, probiotics may have therapeutic potential by inhibiting inflammatory responses, maintaining the gut barrier, and modulating lipid metabolism. The use of probiotics to maintain as well as reshape the balance of GM is an important development in the treatment of cardiovascular diseases such as AS. However, it should be noted that studies on probiotics for the treatment of AS are still relatively limited and the results are inconsistent. Due to species diversity and individual differences, as well as the survival and stability of probiotics, further breakthroughs are needed. In addition, dietary fiber intake and application of natural products are also potential options for modulating the GM to intervene in AS. Natural products, usually of plant and animal origin, have a wide range of diverse biological activities and pharmacological effects and have a wide range of applications and research values in the medical field. In the treatment of AS, natural products have shown great potential (Cheng et al., 2022). The search for effective new drugs from natural products and extracts for the treatment of AS has become a hot research topic in recent years. Natural products, probiotic interventions, fecal microbial transplants, and other therapeutic modalities based on the regulation of intestinal microecology have been proposed and some have been successfully applied in the treatment of AS (Ji et al., 2020; Kim et al., 2022). The advantages of natural products, such as wide sources, low toxicity, and diverse immunomodulatory activities, suggest that natural products have a promising future in the treatment of AS. Through in-depth research on natural products, we can search and discover new compounds with biological activities, which can not only expand the application fields of natural products but also meet human needs for treating diseases and improving the quality of life. Although natural products have shown convincing results in several studies as AS therapies. However, there is still a lack of research on the effects of natural products on the immune system through GM and their metabolites. As mentioned above, in terms of modulating the immune response, current studies have focused on how GM and its metabolites promote or inhibit the development of AS by regulating macrophage infiltration, polarization, cholesterol metabolism, and the associated inflammatory response. There are few studies on how GM affects other immune cells (such as T cells, B cells, mast cells, *etc.*). In addition, the mechanism of production of metabolites associated with GM is unclear, such as how bacteria use dietary fiber to produce SCFAs. This is a gap that needs to be filled in future research. Further research is needed to improve the use of natural products in the treatment of AS.

In addition, there are some limitations in the current studies on the relationship between GM and the immune system, which need further research and exploration. Although many studies have shown that there is an interaction between GM and the immune system, the correlation and causality have not been clearly explained. Most of the current studies are still observational, and it is difficult to determine causality. The interaction between GM and the immune system involves complex biological mechanisms, such as signal transduction, the influence of metabolites, and immune cell interactions. Our current understanding of these mechanisms is relatively limited, and further studies are needed to reveal the details. In addition, there is a lack of consistency in the results obtained from different studies, which may be due to reasons such as study design, sample size, and population differences. There is also the inevitable problem that there are significant differences in GM composition and immune systems between individuals, making the generalization of the findings somewhat challenging.

Future studies should explore the association between different microbiota composition, function, and metabolites and the immune system, as well as their impact on health and disease states, by analyzing large-scale gut microbiota data. In addition, it focuses on the deeper interaction mechanism between gut microbiota and the immune system, including immune regulation, signal transduction, immune cell development, and function. This helps to develop precise regulatory strategies to adjust the structure and function of gut microbiota, thereby affecting the state of the immune system. The differences in gut microbiota and immune system among different individuals cannot be ignored, and their relationship with individual health, susceptibility, and response to treatment can be explored. Considering the great interindividual differences in people’s microbiota composition and immune response, this research can help to achieve personalized medicine, customized nutrition, and treatment programs. In terms of drug development, focusing on how the active components of natural products are metabolized by GM, and whether these metabolites have synergistic or antagonistic effects on atherosclerotic diseases, and then finding new favorable metabolites of GM will provide a new direction for the clinical drug treatment of AS.

In summary, the emerging role of natural products in the treatment of AS still has greater potential and deserves attention. Natural products improve the immune response in AS by modulating the GM and its metabolites, which in turn inhibit the progression of AS. The role of natural products in targeting gut microbiota and immune response provides unique insights into the gut arterial axis and broadens our understanding of natural products.

## Figures and Tables

**Table:** 

**AS**	Atherosclerosis
**LDL**	low-density lipoprotein
**GM**	gut microbiota
**TMAO**	trimethylamine N-oxide
**SCFAs**	short-chain fatty acids
**DCs**	dendritic cells
**ox-LDL**	oxidized low-density lipoprotein
**ROS**	reactive oxygen species
**APCs**	antigen-presenting cells
**CCL**	CC chemokine ligand
**CXCL**	CXC chemokine ligand
**LPS**	lipopolysaccharide
**IL**	interleukin
**cDC**	classical DC
**pDC**	plasmacytoid DC
**Th**	T helper
**TNF-α**	tumor necrosis factor-α
**TGF-β**	transforming growth factor-β
**IFN-γ**	interferon-γ
**TMA**	trimethylamine
**ABCA1**	ATP-binding cassette transporter A1
**VCAM-1**	vascular cell adhesion molecule 1
**HFD**	high-fat diet
**FO B**	follicular B
**MZ B**	marginal zone B
**IRA B**	innate response activator B
**AMPK**	adenosine monophosphate-activated protein kinase
**PSRC 1**	proline and serine-rich coiled-coil protein 1
**SR-B1**	scavenger receptor class B type I
**RCT**	reverse cholesterol transport
**SR-A1**	scavenger receptor scavenger receptor A 1
**FXR**	farnesoid X receptor
**SHP**	small heterodimer partne
**NOS**	nitric oxide synthase
**ApoA-I**	apolipoprotein A-I
**PPARα**	peroxisome proliferator-activated receptor-alpha
**TGR 5**	G-protein-coupled bile acid receptor 5
**RSV**	Resveratrol
**BBR**	Berberine
